# Quantitative Prediction of the Landscape of T Cell Epitope Immunogenicity in Sequence Space

**DOI:** 10.3389/fimmu.2019.00827

**Published:** 2019-04-16

**Authors:** Masato Ogishi, Hiroshi Yotsuyanagi

**Affiliations:** Division of Infectious Diseases and Applied Immunology, The Institute of Medical Sciences Research Hospital, The University of Tokyo, Tokyo, Japan

**Keywords:** T cell epitope, T cell receptor repertoire, immunogenicity, escape mutation, machine learning

## Abstract

Immunodominant T cell epitopes preferentially targeted in multiple individuals are the critical element of successful vaccines and targeted immunotherapies. However, the underlying principles of this “convergence” of adaptive immunity among different individuals remain poorly understood. To quantitatively describe epitope immunogenicity, here we propose a supervised machine learning framework generating probabilistic estimates of immunogenicity, termed “immunogenicity scores,” based on the numerical features computed through sequence-based simulation approximating the molecular scanning process of peptides presented onto major histocompatibility complex (MHC) by the human T cell receptor (TCR) repertoire. Notably, overlapping sets of intermolecular interaction parameters were commonly utilized in MHC-I and MHC-II prediction. Moreover, a similar simulation of individual TCR-peptide interaction using the same set of interaction parameters yielded correlates of TCR affinity. Pathogen-derived epitopes and tumor-associated epitopes with positive T cell reactivity generally had higher immunogenicity scores than non-immunogenic counterparts, whereas thymically expressed self-epitopes were assigned relatively low scores regardless of their immunogenicity annotation. Immunogenicity score dynamics among single amino acid mutants delineated the landscape of position- and residue-specific mutational impacts. Simulation of position-specific immunogenicity score dynamics detected residues with high escape potential in multiple epitopes, consistent with known escape mutations in the literature. This study indicates that targeting of epitopes by human adaptive immunity is to some extent directed by defined thermodynamic principles. The proposed framework also has a practical implication in that it may enable to more efficiently prioritize epitope candidates highly prone to T cell recognition in multiple individuals, warranting prospective validation across different cohorts.

## Introduction

T cell epitopes bound to major histocompatibility complex [MHC; also called the human leukocyte antigen (HLA) in humans] molecules activate T cells to initiate subsequent immunological orchestration (1–3). MHC class I (MHC-I) molecules typically present 8- to 11-aa peptides generated through proteasomal cleavage of intracellular proteins to activate CD8^+^ cytotoxic T lymphocytes (CTLs), whereas MHC class II (MHC-II) molecules with an open-ended binding groove accommodate peptides of more variable length derived from endocytosed proteins to activate CD4^+^ T helper (Th) cells (4). Evidence suggests that not all peptides presented on MHC molecules are immunogenic, i.e., trigger functional T cell activation (5–7). T cells recognize peptide-MHC (pMHC) complexes by their TCRs, most predominantly via complementarity determining region 3 (CDR3) loops (8–10). However, the determinants of epitope immunogenicity in association with their recognition by T cells remain poorly understood. Given the fact that different individuals have different TCR repertoires, in theory, epitope immunogenicity should differ between individuals. However, there are several examples of immunodominant epitopes that are targeted by the adaptive immunity of different individuals (11–15). Indeed, immunodominant epitopes have already been clinically utilized, for example, in the interferon-gamma release assay, a clinically available peripheral blood assay to determine if the subject has previously been sensitized by *Mycobacterium tuberculosis* (Mtb) (16, 17). To explain this phenomenon, it is plausible to hypothesize that those immunodominant epitopes share some intrinsic patterns which render them more prone to be recognized by the T cell immunity of multiple individuals. Because TCR-epitope interaction is governed by the physicochemical principles like other protein-protein interactions, more immunodominant epitopes are expected to have a higher chance of stronger interaction when scanned by a large set of TCRs. In this scenario, we could utilize TCR sequences as “baits” to probe highly immunodominant epitopes. We assumed that commonly shared TCR sequences among multiple individuals (referred to “public TCR repertoire” hereafter) more likely reflect the hidden patterns for immunodominant epitopes. Indeed, contrary to the long-standing view that TCR repertoire is highly stochastic and individualized, accumulating evidence suggests that the biased generation of TCR repertoire due to non-random rearrangement and positive/negative thymic selections leads to an extensive utilization of a limited subset of possible sequences and larger-than-expected overlaps of repertoires across individuals (18–25). Moreover, it has been shown that even distinct TCR repertoires can convergently recognize a limited set of pathogen-derived immunodominant epitopes (12).

In this context, here we propose that a computational framework mimicking the thermodynamic interactions between pMHC complexes and public TCR clonotypes, termed TCR-peptide contact potential profiling (CPP), generates probabilistic estimates of immunogenicity which effectively recapitulate essential characteristics of T cell immunity and enable quantitative assessment of potential mutational impacts on the dynamics of immunogenicity in a position-specific context. Datasets and in-house codes to reproduce the entire work are made available as the package *Repitope* (https://github.com/masato-ogishi/Repitope).

## Results

### The Concept of Repertoire-Wide TCR-Peptide Contact Potential Profiling

**Figure 1:**
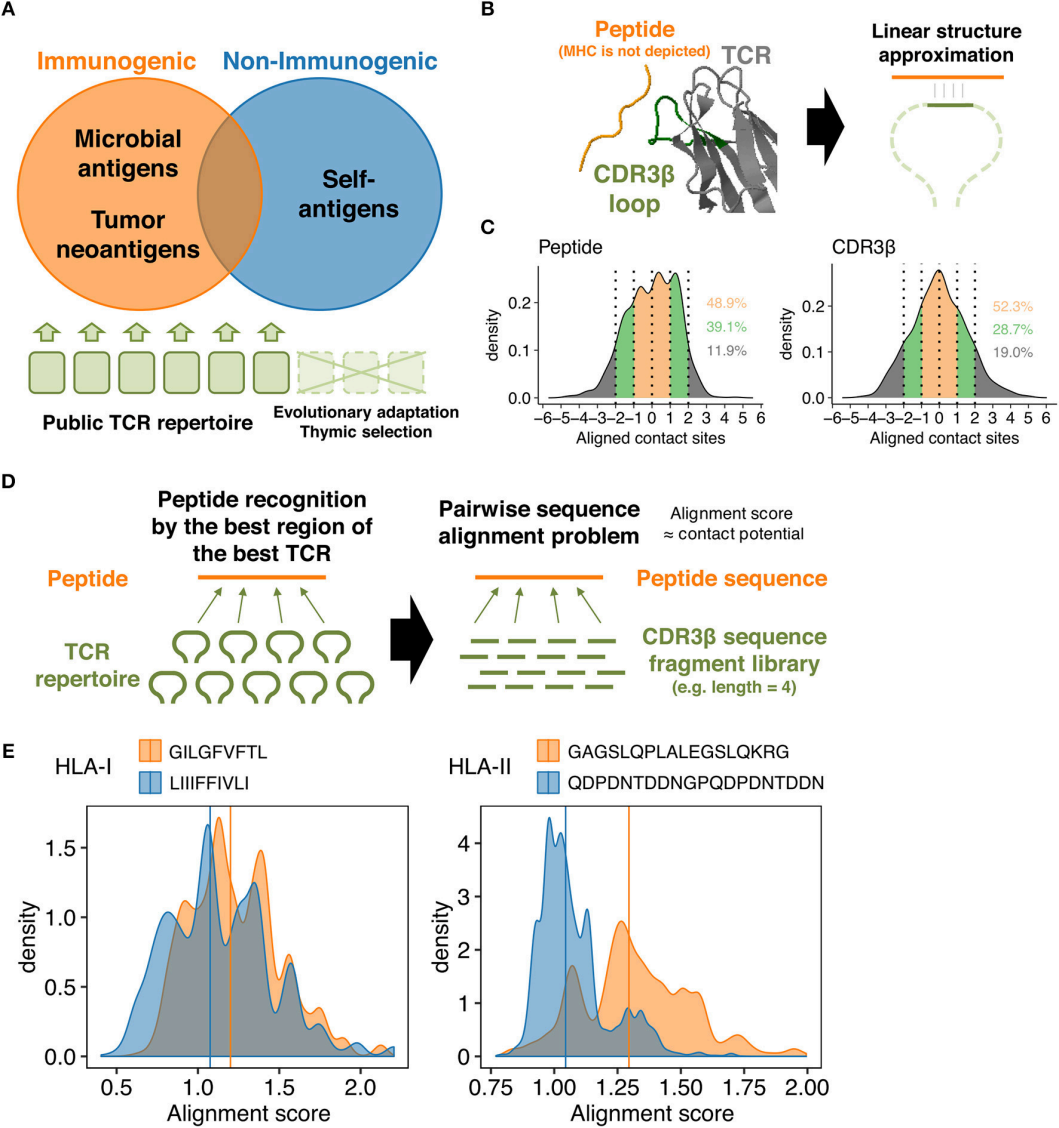
TCR-peptide contact potential profiling. **(A)** Schematic of antigen-guided TCR repertoire convergence hypothesis. The present work is premised on the notion that genome-level evolution and postnatal thymic selection selected certain intrinsic features of TCR repertoire that favor immunodominant epitopes. **(B)** Schematic of the linear contact model. The underlying hypothesis is that only a small portion of the CDR3β loop structure is required (at least initially) to recognize a corresponding epitope. Here, the net energetic potential of the contact is defined as a sum of pairwise amino acid contact potentials of contacting residues. The structure of HIV reverse-transcriptase epitope (TAFTIPSI) and TCR CDR3β chain is shown as an example (PDB ID: 4MJI). **(C)** Mean-centered contact density distributions of 51 unique peptide-TCR structures collected from public sources. Residues were defined in contact if any of their heavy atoms is within a distance of 5.5 Å. Contacting residues were determined using PRODIGY web server (https://nestor.science.uu.nl/prodigy/) (26, 27). Inset numbers indicate the proportions of contacts falling into the color-specified ranges. **(D)** Simplification of the molecular scanning process of a peptide by a given TCR repertoire as a pairwise sequence alignment problem. Pairwise alignment was conducted using custom substitution matrices so that the highest possible alignment score reflect the most robust intermolecular contacts between a peptide and a TCR-derived fragment. An alignment score distribution for any peptide was thus defined using a set of TCR-derived fragments. **(E)** Representative alignment score distributions calculated using 10,000 randomly selected public CDR3β-derived 3-aa fragments and the custom contact potential matrix derived from the AAIndex scale “BETM990101inv.” Pairwise sequence alignment was conducted using the *pairwiseAlignment* function implemented in the *Biostrings* package in R (Methods). Left, two HLA-I-restricted peptides. GILGFVFTL is an immunogenic epitope derived from the matrix protein 1 of influenza A virus. LIIIFFIVLI is a non-immunogenic MHC binder derived from the protein I2 of vaccinia virus. Right, two HLA-II-restricted peptides. GAGSLQPLALEGSLQKRG is an immunogenic self-epitope derived from insulin. QDPDNTDDNGPQDPDNTDDN is a non-immunogenic MHC binder derived from the latent membrane protein 1 of Epstein-Barr virus. Vertical lines indicate the median.

The hypothesis underlying the overall study is that public TCR repertoire is biased toward immunodominant epitopes through evolutional adaptation and negative selection at thymus, and therefore could be used as “baits” to probe peptides with high immunogenic potential (Figure 1A). Because of the sequence-level and structural diversity of TCR and pMHC, it is unfeasible to comprehensibly characterize every single TCR-pMHC interaction experimentally. To circumvent this problem, we instead designed a sequence-based simulation framework designed to mimic molecular scanning of pMHCs by TCR repertoire, which we termed repertoire-wide TCR-peptide contact potential profiling (CPP).

The concept of CPP relies on some simplifications. First, we focused on TCR β chain CDR3 (CDR3β) loops, since this region has the highest genetic variability and is primarily responsible for the interactions with peptides presented onto the MHC grooves, whereas more conserved CDR1 and CDR2 loops typically interact with MHC α-helices (2, 3, 28, 29). Second, we assumed that the recognition of MHC-presented peptides requires only a small portion of the CDR3β loop, at least initially, that could be approximated to linear structure (Figure 1B). This assumption is based on the relatively low affinities (*K*_*D*_ = 0.1 ~ >500 μM) of TCR-peptide interactions compared to other immunoglobulin-like molecules including antibodies, the relatively flat interfaces observed in known TCR-peptide-MHC structures, and the substantial conformational changes induced upon recognition of pMHC complexes (30–33). Moreover, analysis of known TCR-pMHC complex structures using PRODIGY (https://nestor.science.uu.nl/prodigy/) (26, 27) revealed that around 50% and more than 80% of the intermolecular contacts are limited within the 3-aa and 5-aa ranges, respectively, in both peptides and CDR3β regions (Figure 1C and Supplementary Data Sheet 1).

Based on these premises, we first defined the energetic potential of intermolecular contacts between a portion of a peptide and a fragment of a TCR CDR3β region as the sum of amino acid pairwise contact potentials (Figure 1B). Next, we approximated the “best-match” problem between a peptide and the entire TCR repertoire to the pairwise sequence alignment problem, where a set of fragments derived from a given TCR repertoire are pairwise-aligned to the peptide sequence to identify the “best-aligned” positions that maximize their alignment scores (Figure 1D). Usually, a pairwise sequence alignment algorithm attempts to maximize the alignment score that reflects sequence homology. However, we utilize custom substitution matrices during the alignment process so that the alignment scores reflect the energetic potentials of intermolecular contacts (Methods). To put it briefly, higher alignment scores are considered a hallmark of epitope immunogenicity, as exemplified in Figure 1E. For more accurate immunogenicity prediction through machine learning, we defined CPP features as a set of descriptive statistics (e.g., mean and standard deviation) of the alignment score distribution (Methods).

### Datasets and Analysis Workflow

**Figure 2:**
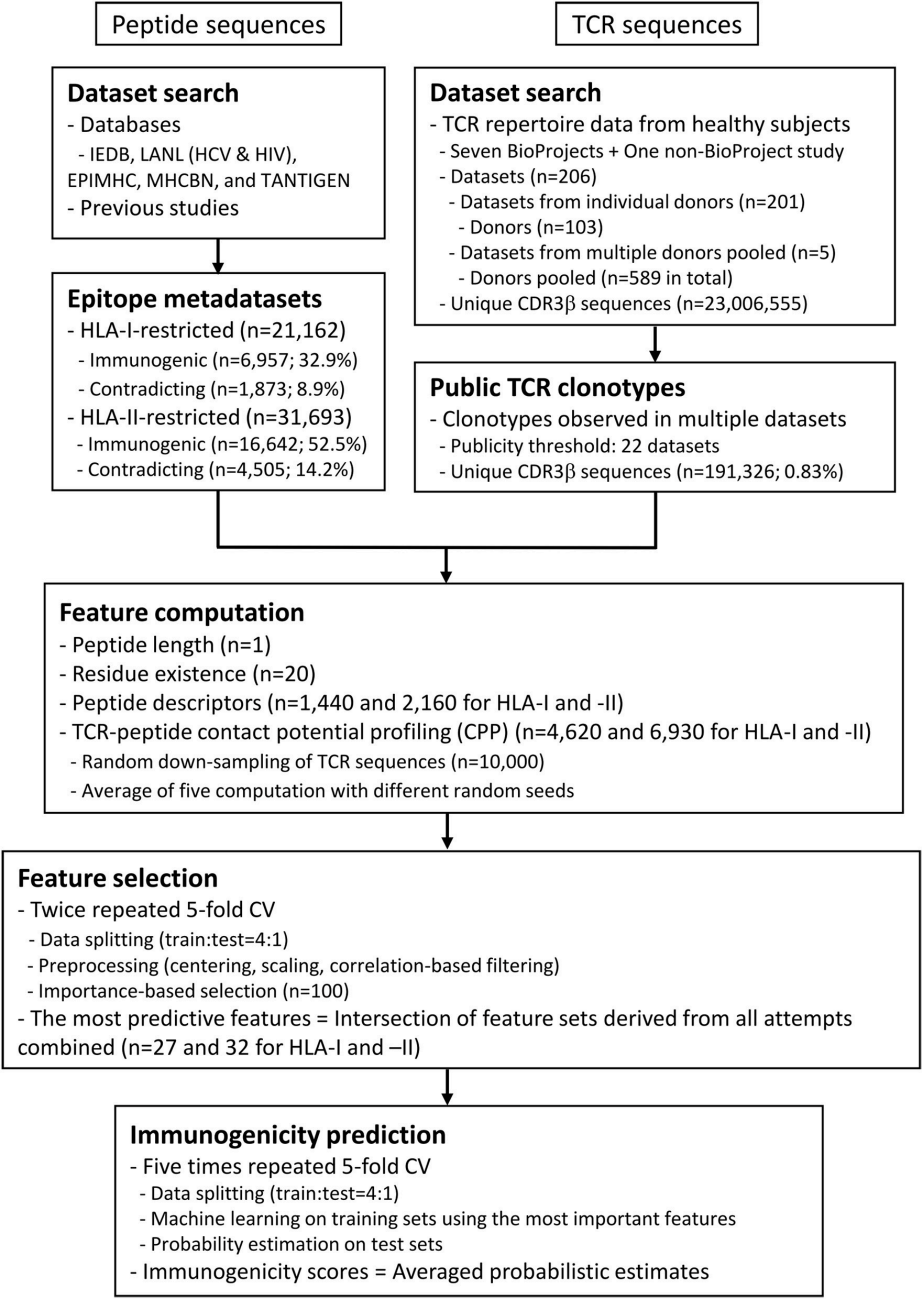
Summary of datasets and analysis workflow. The flowchart describes the outline of data compilation and subsequent computation of the probabilistic estimates of epitope immunogenicity, termed immunogenicity scores. Briefly, T cell epitope sequence data annotated with either *in vitro* or *in vivo* assay results (positive or negative) were retrieved from various public databases. Peptide data were not necessarily accompanied by the restricting MHC metadata. In the meantime, public TCR sequence data were extracted from previous studies. A set of features were computed for each of the peptides, including the CPP features (see also Figure 1 and Methods). Minimal sets of the most predictive features were selected through the feature selection process through twice repeated 5-fold cross-validation (CV). In other words, the entire dataset was split into five chunks, and machine learning was conducted on a leave-one-chunk-out basis. Feature importance was estimated, and the top 100 features were retained. The consensus of the five feature sets was retained. The entire process was repeated twice, and the final consensus of the feature sets was identified as the most predictive features. Finally, probabilistic estimates of immunogenicity were computed through five times repeated 5-fold CV only using the most predictive features. This procedure ensures that an immunogenicity score for any peptide is an average of five machine-learned classifiers trained without using the target peptide itself.

We compiled epitope datasets comprising 21,162 8- to 11-aa HLA-I-restricted and 31,693 11- to 30-aa HLA-II-restricted peptide sequences from various sources (Figure 2, Supplementary Figure 1 and Supplementary Data Sheet 2) (34–42). At this point, we did not filter peptide sequences based on their sequence-level homologies, because even a single substitution could considerably affect their immunogenicity. Annotations of qualitative T cell assay results (i.e., positive or negative) were retrieved from source databases and coalesced by individual peptides. For those obtained from the Immune Epitope Database (IEDB), for which more detailed annotations about the evidence of T cell reactivity were available, only functional cell-based assay results were considered (Methods). Peptides retrieved from the Los Alamos National Laboratory (LANL) HIV and HCV databases and the TANTIGEN database were assumed to be positive *a priori*. In this manner, 21,162 and 31,693 HLA-I and HLA-II-restricted peptides were identified, respectively, of which 1,873 (8.9%) and 4,505 (14.2%) had contradicting annotations on immunogenicity. In such cases, peptides with at least one positive annotation were considered immunogenic, given that coexistence of negative assay results does not necessarily preclude the possibility of being recognized by any of the TCRs in the public TCR repertoire. Eventually, 6,957 (32.9%) and 16,642 (52.5%) HLA-I and HLA-II-restricted peptides were considered immunogenic, respectively. Collected peptide sequences and annotations are summarized in Supplementary Data Sheet 2.

Next, we extracted 23,006,555 unique CDR3β sequences from TCR repertoire datasets derived from healthy individuals using MiXCR software (43). We obtained 191,326 unique CDR3β sequences identified in at least 22 out of 206 (~11%) different datasets. Note that due to limited annotation we were not able to strictly select one repertoire dataset per one donor. However, the aim of collecting CDR3β sequences from multiple datasets is to delineate a public TCR repertoire and use that repertoire as a “bait” for epitope immunogenicity. Therefore, we proceeded to the computation of CPP features using the 191,326 CDR3β sequences.

### TCR-Peptide Interaction Parameters Explaining Epitope Immunogenicity and TCR Affinity

**Figure 3:**
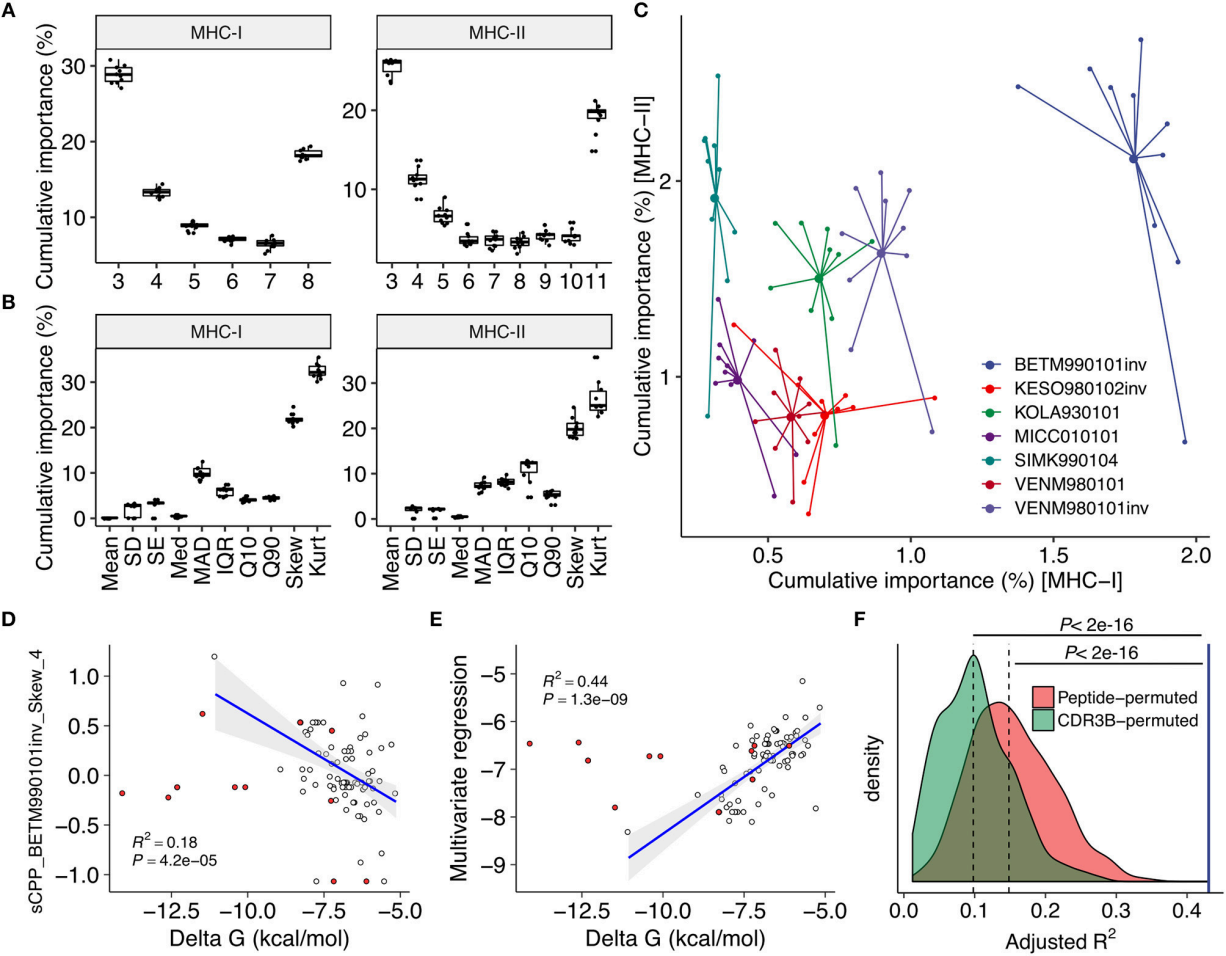
Contact potential profiling parameters linked epitope immunogenicity and TCR affinity. **(A–C)** Feature importance estimates through the feature selection process (see also Figure 2). Estimates from twice repeated 5-fold CV were aggregated. **(A,B)** Importance estimates for all CPP features stratified by **(A)** fragment length and **(B)** descriptive statistics to summarize the distribution of alignment scores (see also Figure 1E and Methods). **(C)** Importance estimates for the most predictive 21 and 26 CPP features for MHC-I and MHC-II, respectively, stratified by their source AAIndex scales. The seven AAIndex scales commonly utilized in the most predictive features for MHC-I and MHC-II are shown. Larger dots indicate means. **(D–F)** Regression analysis against individual TCR affinities. Single-TCR contact potential profiling (sCPP) was conducted for 82 wildtype (WT) TCR-peptide complexes with known affinities identified through literature search (Supplementary Data Sheet 1). Thirteen mutant (MT) TCRs were excluded from correlation analysis but shown for visual comparison purposes (red points). Adjusted squared Pearson's correlation coefficients are presented. **(D)** Representative univariate regression using the top sCPP feature “sCPP_BETM990101inv_Skew_4.” Note that CPP and sCPP feature names are designed to indicate (i) the AAIndex scale, (ii) the descriptive statistics, and (iii) the length of the TCR-derived fragments. **(E)** Multivariate regression with five sCPP features derived from immunogenicity-predicting AAIndex scales. **(F)** Permutation experiments with 1,000 iterations. Multivariate regression was performed as in **(E)** using sCPP features computed from either randomly permuted peptide sequences or CDR3β sequence fragments (Methods). The blue vertical line represents the result of the non-permuted regression analysis. Dashed lines represent medians.

We computed CPP features as well as other features based on peptide physicochemical properties. The 32 and 27 most reproducibly selected features for MHC-I and MHC-II predictions, respectively, were identified (Figure 2, Supplementary Figure 2 and Supplementary Data Sheet 3). Only nine of 32 and none of 27 features were derived from peptide physicochemical descriptors, highlighting the indispensable contributions of CPP features on immunogenicity prediction. Common parameter usage patterns for CPP features were observed in MHC-I and MHC-II (Figures 3A–C). Notably, features derived from short fragments (i.e., 3-aa and 4-aa) and the longest fragment (i.e., 8-aa and 11-aa in MHC-I and MHC-II, respectively) appeared heavily weighted (Figure 3A). Meanwhile, skewness- and kurtosis-derived features showed markedly higher importance values, indicating that distinct repertoire-wide contact potential distributions are the hallmark of immunogenicity (Figure 3B). The inverted version of BETM990101 (termed “BETM990101inv”) (44) and other six AAIndex scales of the highest importance for both MHC-I and MHC-II (Figure 3C).

To test if the selected AAIndex scales reflect thermodynamic aspects of TCR-peptide interactions, we next analyzed 82 TCR-peptide complexes with experimentally determined affinities collected from literature (Supplementary Data Sheet 2) through a modified framework termed single-TCR contact potential profiling (sCPP). We found that BETM990101inv and other six AAIndex scales important for immunogenicity prediction also generated sCPP features correlating with affinities (Figures 3D,E and Supplementary Table 1). As expected, multivariate regression did not find any correlation between affinity and sCPP features computed if either peptide or TCR sequences were permuted before sCPP feature computation, indicating the importance of sequence-intrinsic hidden patterns (Figure 3F). Collectively, these observations support the idea that our CPP framework recapitulates the essential thermodynamic properties of TCR recognition of pMHC complex which governs both MHC-I and MHC-II systems.

### Probabilistic Estimation of Epitope Immunogenicity

**Figure 4:**
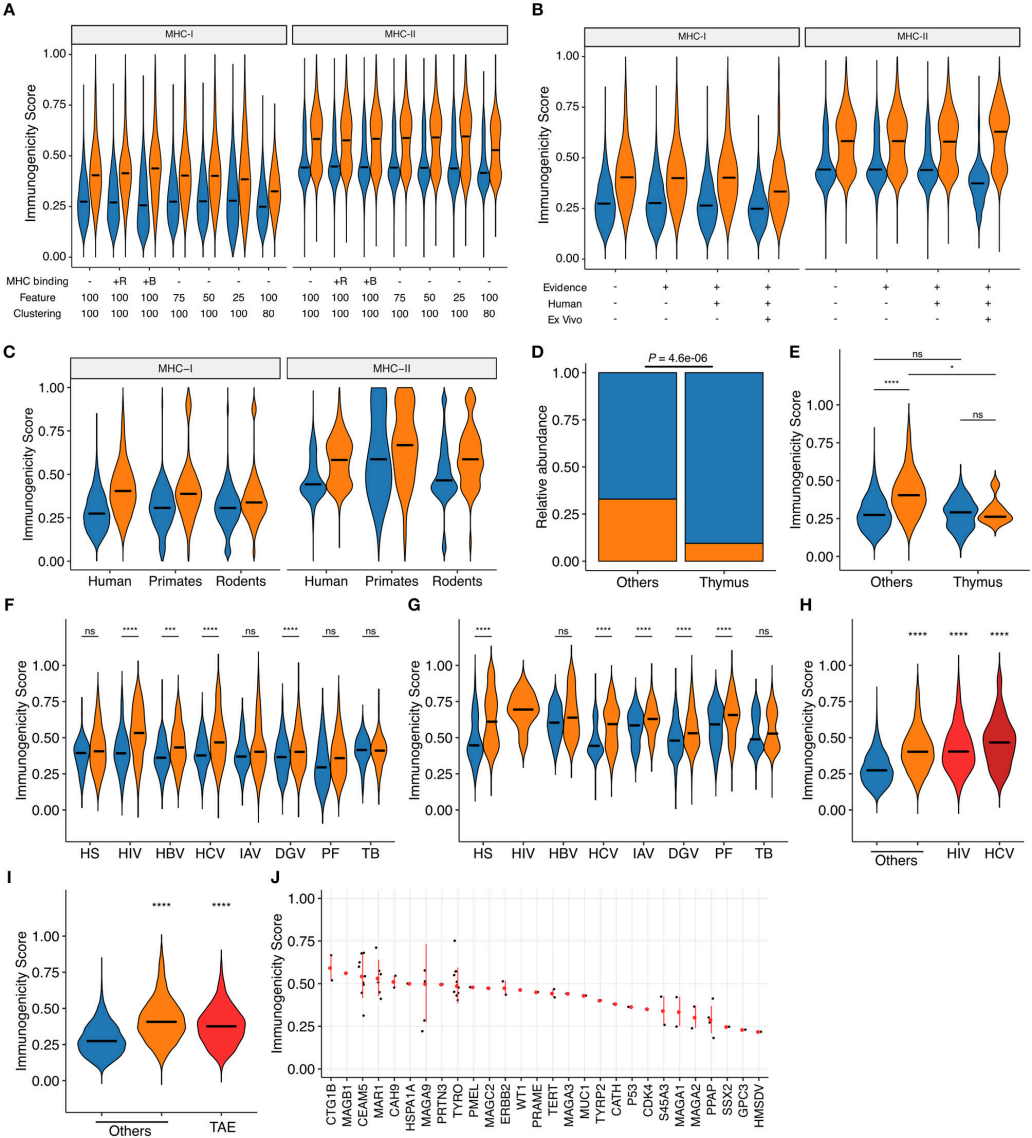
Probabilistic prediction of epitope immunogenicity. Averaged probabilistic estimates from iterative machine learning were termed “immunogenicity scores.” Throughout the figure, orange and blue represent immunogenic epitopes and non-immunogenic MHC binders, respectively, as in Figure 1A. For the definition of immunogenicity, see the main text and Methods. **(A)** Comparison of probabilistic estimates generated through machine learning in various settings. MHC binding: MHC binding predictions were incorporated as features in addition to the default CPP and peptide features for machine learning. +R, percentile ranks; +B, binding strength categories. Feature: the cutoff number for feature selection process was changed as indicated. The numbers of features retained after feature selection under the cutoff of 100, 75, 50, and 25 were 32, 26, 19, and 7 for MHC-I, and 27, 23, 21, and 12 for MHC-II, respectively. Clustering: highly homologous peptides were eliminated before machine learning using the IEDB Epitope Cluster Analysis Tool (http://tools.iedb.org/cluster/) with the sequence-level homology threshold of either 100% (i.e., no homology-based peptide removal) or 80%, where one peptide per each homology-based cluster was randomly chosen. The numbers of peptides retained after 80% homology clustering were 16,765 and 23,983 for MHC-I and MHC-II, respectively. **(B)** Immunogenicity scores generated for various subsets of our epitope datasets. Machine learning was conducted with 32 and 27 pre-selected features for MHC-I and MHC-II, respectively, but without predicted MHC binding information. Evidence: IEDB-derived peptides with functional T cell assay evidence (i.e., epitopes from non-IEDB sources lacking T cell assay annotation were excluded); Human: epitopes tested in human (i.e., epitopes tested only in transgenic animal models were excluded); *ex vivo*: IEDB-derived peptides annotated from direct *ex vivo* assays using autologous effector and antigen-presenting cells in human. The numbers of peptides included after applying each filter were 21,162, 13,058, 11,084, and 6,048 for MHC-I, and 31,693, 31,693, 30,188, and 2,023 for MHC-II, respectively. **(C)** Immunogenicity scores of human epitopes and extrapolated scores for primate and rodent epitopes (*N* = 411 and 8,756 for MHC-I and 76 and 8,445 for MHC-II, respectively). **(D,E)** Distributions of **(D)** annotated immunogenicity and **(E)** immunogenicity scores for the human thymus MHC-I peptidome (45). **(F,G)** Immunogenicity scores among IEDB-derived **(F)** MHC-I- and **(G)** MHC-II-restricted peptides of various origins. HS, *homo sapiens*; HIV, human immunodeficiency virus type I; HBV, hepatitis B virus; HCV, hepatitis C virus; IAV, influenza A virus; DGV, dengue virus; PF, *Plasmodium falciparum*; TB, *Mycobacterium tuberculosis*. **(H)** Immunogenicity scores among MHC-I-restricted peptides from various sources. HIV, Los Alamos HIV Epitope Database; HCV, Los Alamos HCV Epitope Database. **(I,J)** Immunogenicity scores of MHC-I-restricted tumor-associated epitopes (TAEs) identified from TANTIGEN database. In **(J)**, TAEs were grouped by their source tumor antigens. Red points and bars represent median and interquartile ranges, respectively. In **(A–D)** and **(F–H)**, bars represent medians. In **(E–I)**, ns, not significant; ^*^P < 0.05; ^***^P < 0.001; ^****^P < 0.0001.

We applied machine learning techniques to convert these most predictive features into a unidimensional scale of immunogenicity by averaging the probability estimates from five times repeated 5-fold cross-validations (CVs). The estimates were reasonably consistent across CVs; the normalized standard deviations distributed with medians and interquartile ranges (IQRs) of 8.3% (2.3 to 14.3%) and 5.0% (1.4 to 8.5%) in MHC-I and MHC-II, respectively. We first tested several settings for machine learning. Addition of MHC binding prediction results as features for machine learning only marginally improved the predictive performance for MHC-I and had almost no effect for MHC-II. Reduction of the number of features utilized during machine learning and removal of peptides with high sequence-level homology (i.e., 80% or higher) from the dataset before machine learning resulted in only a modest decrease in predictive performance (Figure 4A and Supplementary Figure 3). Based on these observations, we decided to utilize the averaged probabilistic estimates derived without MHC-associated features hereafter (termed as “immunogenicity scores”) (Supplementary Data Sheet 4). Next, we examined whether immunogenicity scores were applicable to the entire dataset. Indeed, there was an apparent separation of immunogenic epitopes and non-immunogenic MHC binders across various subsets of the datasets. This was indeed the case even when we focused on the epitope data obtained from *ex vivo* assays using human subject-autologous antigen-presenting cells and T cells (Figure 4B). Then, we examined the applicability and implication of the immunogenicity score system in various biological contexts. First, immunogenicity score framework was extrapolated to non-human peptide datasets by applying models trained from the human peptide datasets. Overall, the classification performance of extrapolated immunogenicity scores less efficiently distinguished immunogenic epitopes from non-immunogenic binders (Figure 4C and Supplementary Figure 4). Second, thymically expressed self-epitopes obtained from a previous study (45) were compared to the rest of the peptides. Interestingly, based on database annotation, the ratio of immunogenic epitopes was lower among thymic self-peptides than the rest of the peptide data. In addition, even peptides annotated as immunogenic had overall lower scores than non-thymus immunogenic epitopes, but comparable scores to thymically expressed non-immunogenic peptides (Figures 4D,E). Third, immunogenic peptides from various organisms generally had higher scores than non-immunogenic counterparts, with some exceptions such as MHC-I peptides derived from human and Mtb. Meanwhile, immunogenicity scores fit particularly well into IEDB-derived HIV and HCV peptides (Figures 4F,G), and this was also the case in the epitopes from the Los Alamos HIV and HCV immune epitope databases (Figure 4H). Likewise, MHC-I-restricted tumor-associated epitopes obtained from TANTIGEN database also exhibited higher scores than non-immunogenic peptides from various origins (Figure 4I). Tumor-associated antigens yielding peptides of high predicted immunogenicity included CTG1B and MAGB1 (Figure 4J). As a comparison, we tested the MHC-I immunogenicity prediction tool currently available in IEDB (http://tools.iedb.org/immunogenicity/) (34) using the same MHC-I epitope dataset but only found marginal predictive power (Supplementary Figure 5). Note that there is no tool available for MHC-II immunogenicity prediction to compare. Collectively, immunogenicity scores are the quantitative metric of the probability of functional T cell recognition, and would provide another layer of criteria for robust prioritization of MHC-I and MHC-II epitopes with high expected immunogenicity.

### Dynamics of Epitope Immunogenicity by Single Amino Acid Alterations

**Figure 5:**
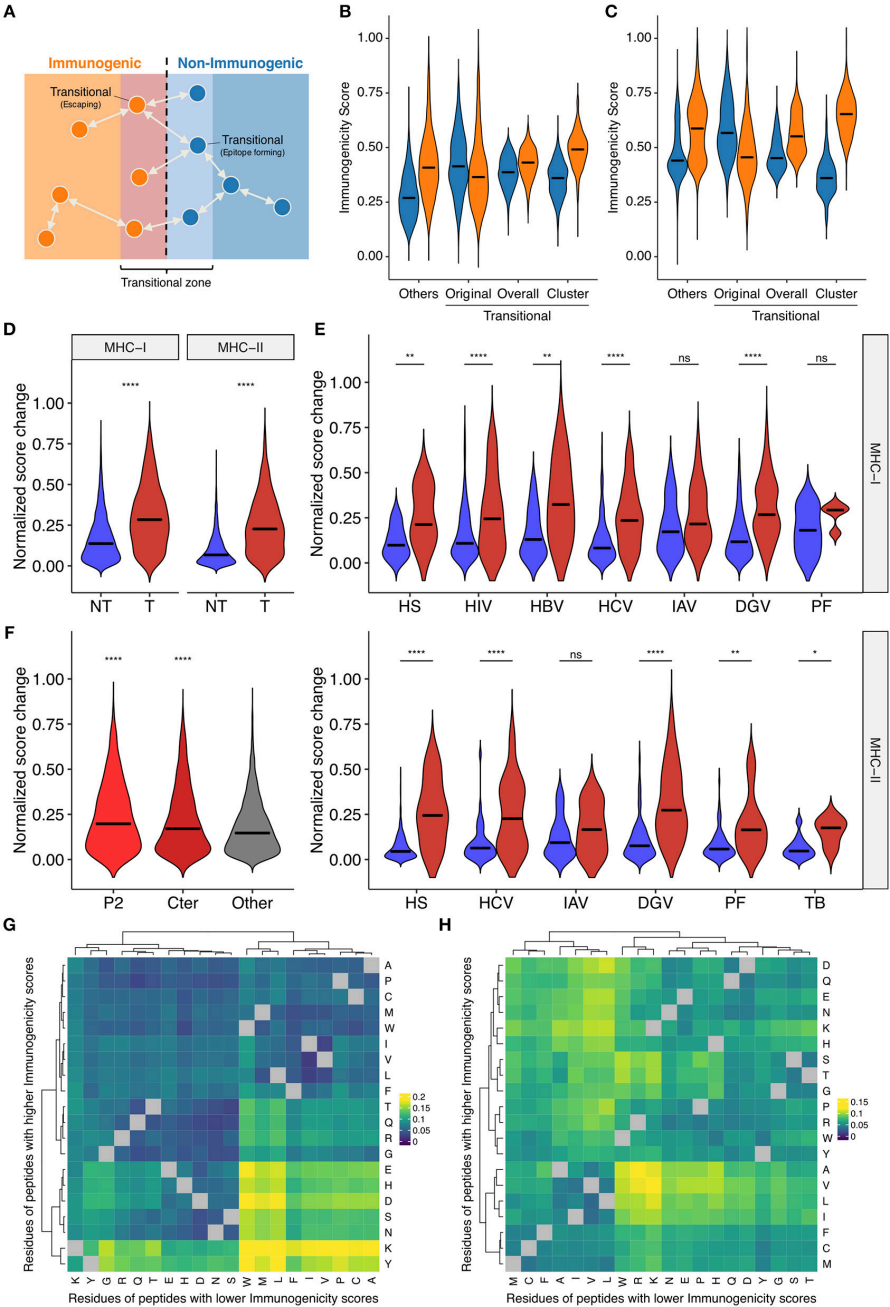
Systematic characterization of the impacts of single amino acid mutations on immunogenicity in sequence space. **(A)** Schematic of the concept of immune transition in sequence space. We termed the change in immunogenicity between neighbors, i.e., single-aa mutants, as “immune transition,” and defined peptides with evidence of immune transition as “transitional.” **(B,C)** Immunogenicity scores among **(B)** MHC-I- and **(C)** MHC-II-restricted transitional peptides, i.e., peptides with at least one neighbor of opposite immunogenicity annotation in our dataset. We identified 1,360 and 976 transitional peptides for MHC-I and MHC-II, respectively. We expanded their neighbor networks by computing immunogenicity scores for 232,723 and 292,437 all possible single-aa mutants of transitional MHC-I- and MHC-II-restricted peptides, respectively. Original, original immunogenicity scores; Overall, mean immunogenicity scores of all neighbors. Cluster, mean immunogenicity scores of neighbors assigned to the cluster containing the parent peptide. Others, original immunogenicity scores for peptides with no evidence of immune transition (shown for comparison). Orange and blue denote immunogenic and non-immunogenic peptides, respectively. **(D–F)** Immunogenicity score dynamics among observed neighbor pairs. We identified 6,179 and 3,076 single-aa mutated peptide pairs for MHC-I and MHC-II, respectively. **(D)** Score dynamics for all possible peptide pairs, regardless of their origins. **(E)** Score dynamics for pairs of peptides derived from the same organism. Blue and red denote non-transitional and transitional pairs, respectively. **(F)** Score dynamics of MHC-I peptide pairs stratified by their mutated positions. Cter, C-terminal residue. **(G,H)** Heatmap clustering analysis of immunogenicity score dynamics by mutating residue pairs. We generated 2,580,890 and 3,101,092 neighbor pairs from 232,723 to 292,437 all possible single-aa mutants of 1,360 and 976 transitional MHC-I- and MHC-II-restricted peptides, respectively. Color represents median normalized score changes. Gray indicates unobserved residue pairs. In **(D–F)**, ns, not significant; ^*^P < 0.05; ^**^P < 0.01; ^****^P < 0.0001.

Single amino acid mutations can affect peptide immunogenicity bidirectionally, namely, acquisition and loss of immunogenicity, in sequence space (Figure 5A). We termed the discordance of annotated immunogenicity between neighbors, i.e., single-aa mutants, as “immune transition,” and defined peptides with evidence of immune transition as “transitional.” Note that we cannot confidently define “non-transitional” peptides solely based on our datasets, because the lack of evidence of immune transition could simply be the lack of experimental data. We identified 1,360 and 976 transitional peptides for MHC-I and MHC-II, respectively. We noted that immunogenicity scores were less applicable to transitional peptides (Figures 5B,C and Supplementary Figure 6), which may result from suboptimal machine learning due to the dearth of transitional peptide data. To test if the integration of neighbor information improves immunogenicity prediction for transitional peptides, we next constructed neighbor networks for transitional peptides using all possible single-aa mutants simulated computationally (*N* = 232,723 and 292,437 for MHC-I and MHC-II, respectively), and compute immunogenicity scores by extrapolation. We termed this process as “*in silico* mutagenesis.” As expected, the averaged extrapolated immunogenicity scores more accurately predicted the immunogenicity of transitional peptides (Figures 5B,C). To further characterize the position- and residue-specific mutational impacts, we next focused on the immunogenicity dynamics between neighbor pairs. We screened our datasets to identify 6,179 and 3,076 MHC-I and MHC-II-restricted single-aa mutated peptide pairs. Immunogenicity scores changed more dynamically in transitional pairs (Figure 5D). This observation was also the case when we focused on the neighbor pairs of the same organismal origins (Figure 5E). We also noted that scores changed more eminently among MHC-I-restricted neighbor pairs with mutations in their anchor residues (Figure 5F). This is an interesting observation since our framework does not utilize any positionally defined features. To gain insights into the residue-specific mutational impacts, we next analyzed 2,580,890 and 3,101,092 neighbor pairs from all simulated single-aa mutants of transitional MHC-I- and MHC-II-restricted peptides, respectively (Figures 5G,H). We utilized simulated peptide data because neighbor pairs identified in experimentally tested epitope datasets were too sparsely distributed and likely biased. Heatmap clustering analysis revealed residue-specific impacts on immunogenicity that are partially interpretable with known physicochemical properties of amino acid residues [e.g., hydrophobic/aliphatic (V, L, and I), negative (D and E), and aromatic (F and W)]. However, some exceptions were also notable [e.g., positive (K and R) in MHC-I]. Collectively, our systematic characterization delineates the landscape of bidirectional effects of single-aa mutations on immunogenicity in a position- and residue-dependent context.

### Inference of Escape Mutations From Simulated Neighbor Network Architecture

**Figure 6:**
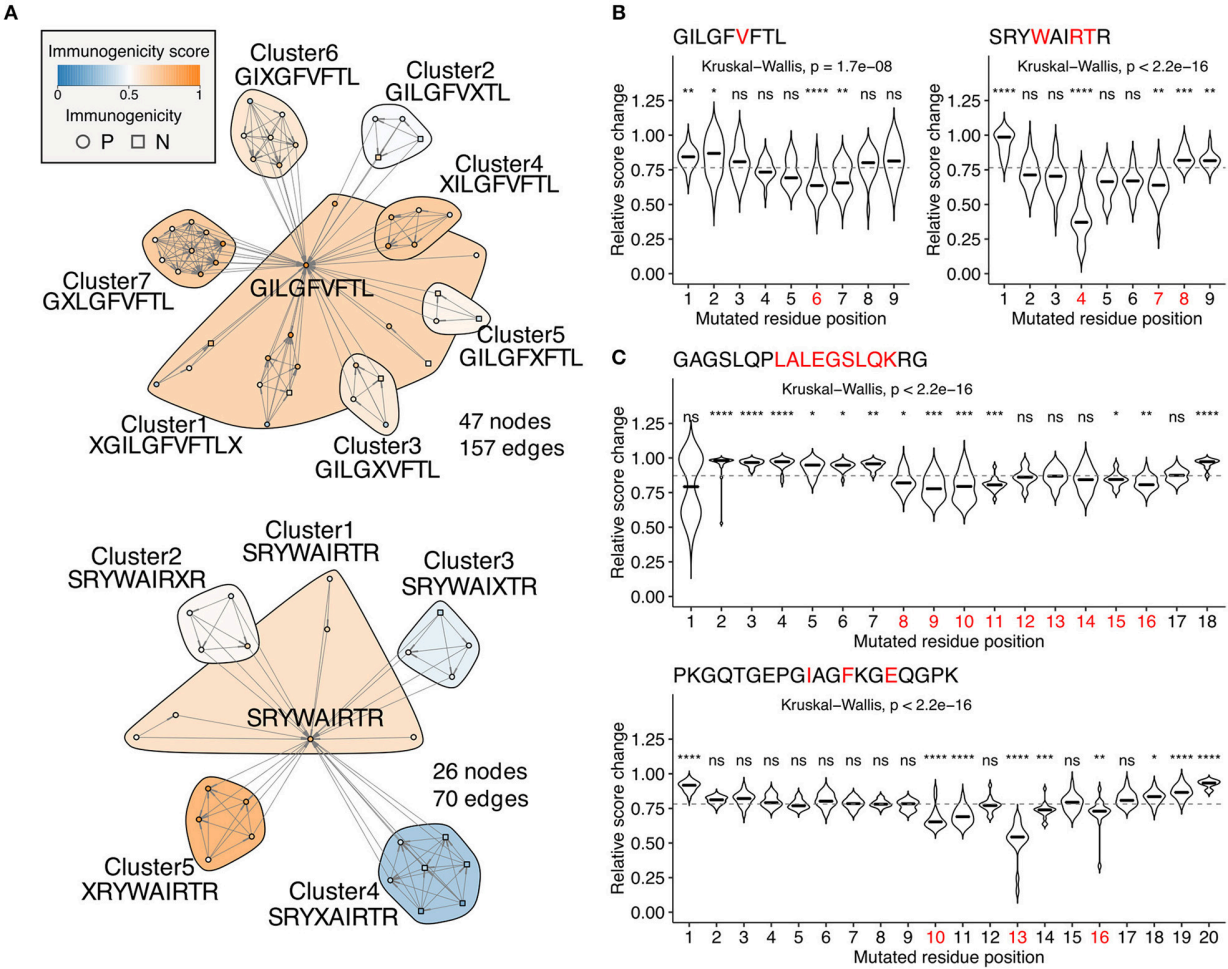
Identification of escape-prone positions from *in silico* mutagenesis and neighbor network analysis. **(A)** Example neighbor networks of MHC-I-restricted influenza A virus epitopes GILGFVFTL and SRYWAIRTR and their observed neighbors. Consensus sequences are indicated below the cluster IDs. **(B,C**) Relative score changes estimated from all possible simulated neighbors for **(B)** MHC-I-restricted influenza A virus epitopes GILGFVFTL and SRYWAIRTR and **(C)** MHC-II-restricted insulin-derived epitope GAGSLQPLALEGSLQKRG and collagen-derived epitope PKGQTGEPGIAGFKGEQGPK. Red letters indicate residues known to have escape mutations shown by *in vitro* T cell assays from the literature. Representative peptides were chosen based on their high immunogenicity scores with manual literature inspection. ns, not significant; ^*^P < 0.05; ^**^P < 0.01; ^***^P < 0.001; ^****^P < 0.0001.

Escape mutations are the principal obstacle for vaccine development. Neither reactivity of the specific T cell clones tested *in vitro* against target epitopes or immune activation after administration *in vivo* does not guarantee a sustainable immune response in the real-world setting because of the possibility of deleterious escape mutations. We tackled this fundamental problem by quantifying position-specific “escape potentials” from neighbor network architecture. Figure 6A shows two representative neighbor networks constructed from observed neighbors of immunodominant influenza A virus epitopes GILGFVFTL (GIL) and SRYWAIRTR (SRY). Apparently, GIL seems more robustly immunogenic than SRY because the Cluster4 in the SRY network was enriched with non-immunogenic peptides, suggesting a path for escaping. *In silico* mutagenesis followed by neighbor network clustering analysis revealed position-specific dynamics of predicted immunogenicity (Figure 6B). P6 and P4 were indicated to have the most substantial impact on escaping for GIL and SRY, respectively. Intriguingly, P6 of GIL peptide has been shown to be involved in two hydrogen bonds with two distinct TCR clones F50 and JM22 (46). Furthermore, five P4 mutants of SRY peptide have been shown to result in an undetectable level of cytolysis by at least three CTL clones (47). Encouraged by these observations, we next examined two MHC-II-restricted epitopes. GAGSLQPLALEGSLQKRG (GAG) is a self-epitope derived from insulin. Our simulation indicated that P8-11 and P15-16 were required for its immunogenicity. Strikingly, immunodominant epitope LALEGSLQK has been localized previously, showing a complete match with the prediction (48). It is worth mentioning that LALEGSLQK is usually degraded proteolytically during the maturation of the insulin molecule. Since our framework does not integrate factors affecting intracellular peptide processing and presentation, appropriate peptide presentation onto MHC should be considered a prerequisite for performing immunogenicity score-based analysis. PKGQTGEPGIAGFKGEQGPK (PKG) is another self-epitope derived from collagen. The simulation indicated that P10, P11, P13, and less evidently, P14 and P16 were required for its immunogenicity. Indeed, mutations at P10, P13, and P16 have been shown to almost diminish the T cell reactivity collected from pre-immunized transgenic mice, and mutations at P14 partially impaired the response (49).

**Figure 7:**
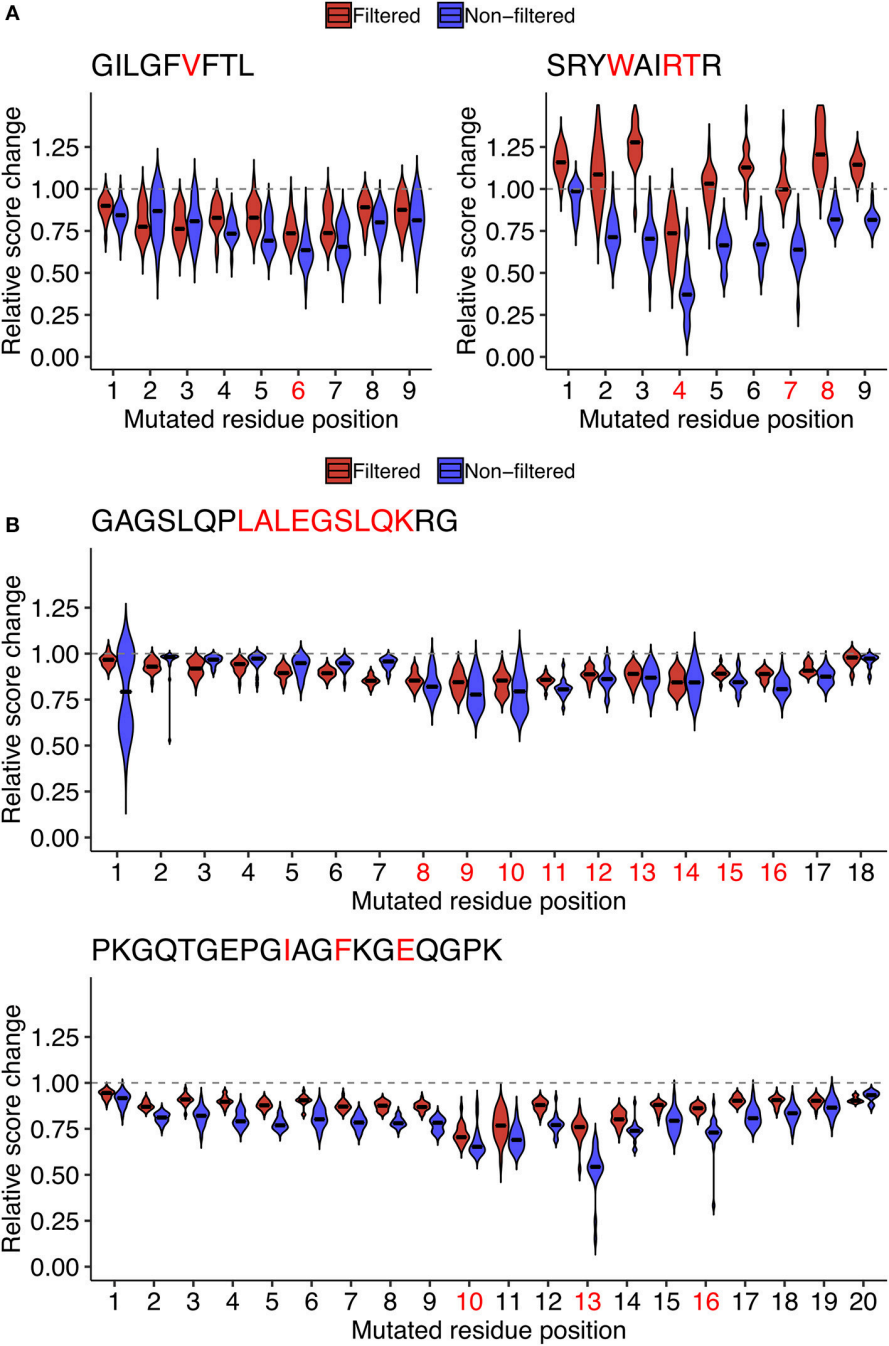
Assessment of the potential impact of overfitting for estimating the position-specific escape potentials. To test if the observed patterns of score dynamics originated from overfitting during the machine learning, for representative **(A)** MHC-I and **(B)** MHC-II peptides shown in Figure 6, probabilistic estimates of immunogenicity were re-computed without using any of the data of the single amino acid variants of the target peptide during machine learning (Red). The scores with no filteration were shown for comparison (Blue). See the legend of Figure 6 for further information.

One possibility is that the epitope- and position-specific dynamics of simulated immunogenicity scores originated from overfitting of machine-learned models to the single amino acid variants of the target peptide of interest in the source dataset. To test this possibility, we repeated the entire analysis from machine learning to the computation of immunogenicity scores to neighbor network simulation using datasets in which both the target peptide of interest and all of its neighbors were removed. Indeed, the most prominent escape-prone positions for every peptide tested, namely, P6 of GIL, P4 of SRY, P8-P14 of GAG, and P10/P13 of PKG, were still identifiable regardless of the presence or absence of neighbor peptides in the source datasets (Figure 7). Meanwhile, it is also notable that some positions such as P7 of SRY and P16 of PKG became less prominent after removal of neighbor peptides from the source datasets, indicating that epitope-specific patterns could also be incorporated from their neighbors as well as some pan-epitope principles of immunogenicity learned from a general set of epitopes.

**Figure 8:**
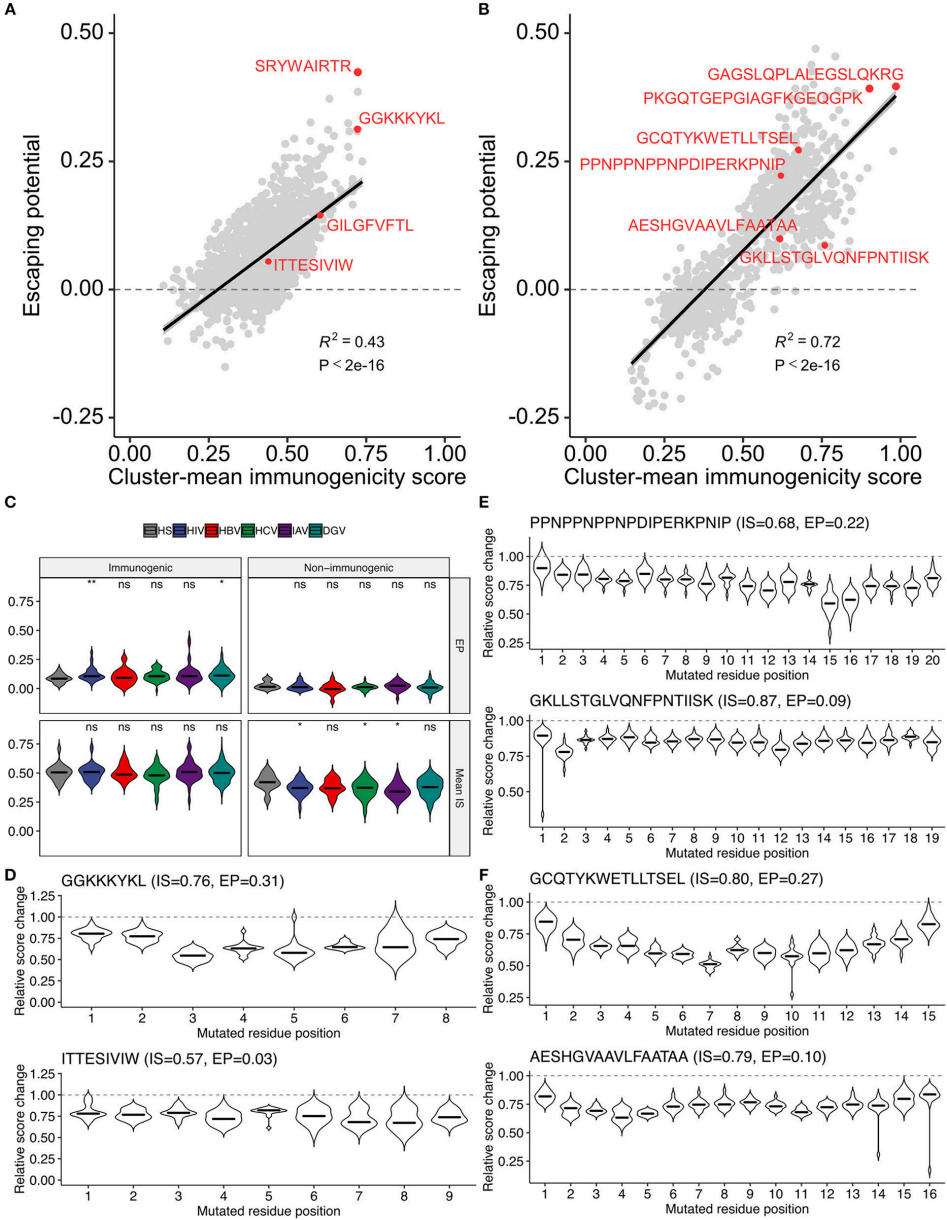
Two-dimensional assessment of epitope quality using the immunogenicity score and escape potential.**(A,B)** Immunogenicity score-escape potential (IS-EP) plots for **(A)** MHC-I and **(B)** MHC-II peptides. Cluster-mean immunogenicity scores determined from neighbor network analysis of all simulated neighbors were utilized. **(C)** IS and EP distributions among pathogen-derived peptides. ns, not significant; ^*^P < 0.05; ^**^P < 0.01. **(D–F)** Relative score changes estimated from all possible simulated neighbors for **(D)** representative MHC-I-restricted HIV epitopes, and **(E,F)** representative MHC-II-restricted epitopes derived from **(E)** *P. falciparum* and **(F)** *M. tuberculosis*. In **(D–F)**, representative peptides were chosen based on their high and low EPs with manual literature inspection.

The methodology to computationally prioritize epitopes of high immunogenicity and low escape potential would have enormous practical implication for developing immunotherapies applicable to global populations. To this end, we introduced another metric termed “escape potential,” which was defined as the maximal difference of cluster-averaged immunogenicity scores between the cluster of interest (containing the target peptide of interest) and other clusters in the neighbor network constructed from all possible single amino acid variants. A negative escape potential means that the target peptide belongs to the least likely immunogenic cluster. Immunogenicity score-escape potential (IS-EP) two-dimensional plots indicated that, although there was a moderate correlation between IS and EP, considerable variation also existed (Figures 8A,B). Of note, HIV and dengue virus (DGV)-derived immunogenic epitopes had higher EP than human epitopes compared, and this trend was not observed in non-immunogenic binders (Figure 8C). Two representative HIV-derived MHC-I epitopes with high (GGKKKYKL) and low (ITTESIVIW) EPs were shown in Figure 8D. Interestingly, there are known naturally occurring escape mutations at P3, P5, and P7 of GGKKKYKL (50), of which P3 and P5 appeared consistent with our analysis (Figure 8D). Although we did not find information on escape variants of ITTESIVIW from the literature, this epitope represents the most common sequence found in the circulating HIV-1 clade B population in the Los Alamos HIV Sequence Database, suggesting that this epitope may be less prone to undergo mutations (51). Likewise, two representative MHC-II epitopes with high and low Eps derived from *P. falciparum* and two from Mtb were shown in Figures 8E,F, respectively. Indeed, the prevalence of T cell reactivity to the low-EP epitope GKLLSTGLVQNFPNTIISK was reported to be 25% in a Kenyan cohort (16). Moreover, 29.4% of South African patients with either latent or active Mtb infection were shown to be positive by ELISpot assay against the epitope VRAVAESHGVAAVLFAATAA, C-terminus of which is identical to the low-EP peptide AESHGVAAVLFAATAA (17). Collectively, these observations indicate that the proposed framework would serve as a valuable tool, along with other bioinformatics tools such as intracellular antigen processing prediction and MHC binding prediction, to screen a large set of epitope candidates before experimental validation based on the two newly introduced metrics, namely, the immunogenicity score and escape potential.

## Discussion

This study provides a novel viewpoint that targeting of epitopes by human adaptive immunity is actually to some extent predictable by defined thermodynamic principles recapitulating the thermodynamic interaction profiles between MHC-presented peptides and the host TCR repertoire. Although previous studies have suggested the relevance of the physicochemical property of TCR-contact residues of the peptides and population-level frequency of peptide-specific T cell clones to epitope immunogenicity (35, 52), this study is to our knowledge the first to illustrate, through leveraging the largest-ever epitope datasets, quantitative prediction of immunogenicity for each individual epitope with sufficiently high consistency with T cell-based assays. However, it should be noted that our framework is not intended for replacing other bioinformatic tools for epitope prediction or experimental testing but rather adding an additional filter to expedite the discovery of potent epitopes. For example, our framework does not take into account the intracellular processing of proteinous antigens, which is taken into account in other tools (53, 54). Moreover, our framework *a priori* takes peptide presentation on MHC for granted; therefore one must test whether the peptides of interest are presented on MHC by either bioinformatic approach such as NetMHC or experimental verification (55, 56). The MHC allele frequency of the target population should also be taken into consideration if population-level immunogenicity is of interest. Finally, experimental testing by comparing with established immunodominant epitopes is indispensable as illustrated by others in search of immunodominant Mtb-derived epitopes (57).

Analysis of contact potential profiling (CPP)-based features predictive of epitope immunogenicity is likely to provide novel clues for describing the molecular scanning process of pMHCs by the host TCR repertoire. It is of note that short (i.e., 3-aa and 4-aa) TCR fragments in combination with a restricted set of AAIndex scales generate features contributing to both MHC-I and MHC-II immunogenicity prediction and also correlating with individual TCR-pMHC affinities because these commonly utilized parameters could potentially reflect some pan-epitope principles governing TCR-pMHC interactions regardless of MHC classes and alleles. The utilization of short fragment features is consistent with the induced fit model that only a limited complimentary region of TCR is involved at least in initial engagement (31–33). Meanwhile, the best AAIndex scale BETM990101 reflects interaction energies between amino acid residues under physiological conditions (44), and hence the inverted version of BETM990101, giving larger values for stronger interresidue contacts, is a reasonable scale for contact potential estimation. Indeed, a positive skewness in the contact potential distribution, implying the coexistence of a small and a large proportion of TCR fragments with high and low contact potentials, respectively, was associated with stronger affinities in our analysis. TCR affinity has been correlated with T cell activation (58), although there remains a debate as to whether other parameters such as avidity, on- and off-rates are also essential (59). Further studies are required to fully elucidate the biological relevance of simulated TCR-peptide contact features quintessential for immunogenicity prediction to the thermodynamic parameters of TCR-pMHC interactions and subsequent T cell activation.

Immunodominance, an immunological phenomenon in which adaptive immunity preferentially targets only a few of the antigenic peptides out of the many others, has been extensively described in multiple cases (15). For instance, only 0.03% of potential MHC-I-presented peptides derived from vaccinia virus account for >90% of the CD8^+^ T cell response in B6 mice (60). Similarly, diverse TCR repertoires specific for two immunodominant viral epitopes have been reported in human (12). Population-level immunity against various immunodominant epitopes is another line of evidence (16, 17). The fact that diverse TCRs can be utilized to achieve protective immunity against immunodominant epitopes in multiple individuals indicates the existence of some epitope-intrinsic qualities guiding “convergent evolution” of human TCR repertoire at both genomic and thymic level. To elucidate the hitherto unknown pan-epitope principles governing productive TCR-peptide contacts, we took an inductive approach using a large set of *in vitro* T cell epitope assay results which may vary depending on the specific T cell clones or TCR repertoires utilized. Our findings indicate that, unexpectedly, a limited set of TCR-peptide contact features reproducibly serve as the principal determinants of T cell reactivity. Moreover, we show that the averaged probabilistic estimates, termed immunogenicity scores, are consistently higher in epitopes with positive results from various functional T cell reactivity assays. Interestingly, peptides physiologically presented on the thymus have lower immunogenicity scores than non-thymus counterparts, which is consistent with the biased formation of human TCR repertoire owing to negative selection (25, 45). Practically speaking, the probabilistic scoring system would be particularly useful for prioritizing peptide candidates to expedite the development of population-level immunotherapies for various infectious diseases and cancer. However, it is notable that immunogenicity scores do not distinguish immunogenic and non-immunogenic peptides from any origin. A particularly notable exception is Mtb; the Mtb epitopes with positive T cell reactivity were assigned with lower immunogenicity scores compared to those from other pathogens, like the thymically-presented self-epitopes with positive T cell reactivity. This observation implies that, from the T cells' point of view, those Mtb epitopes “look like” non-targetable MHC binders like the self-epitopes presented in the thymus. Note that the immunogenicity score is a repertoire-based probabilistic metric, whereas T cell-based assay determines the presence or absence of individual T cell clones reactive of the peptide of interest. Given the ~70,000 years of co-evolution between Mtb and humans (61), one hypothesis is that Mtb has evolutionarily adapted to human T cell immunity to mimic self-epitopes (again, not in terms of sequence-level homology but from “T cells' perspective”), and only highly distinct T cell clones can recognize those Mtb-derived epitopes. Whether these clones function as pro-inflammatory or regulatory is another layer of a question. Notably, it has been shown that T cell epitopes of Mtb are evolutionary hyperconserved and the bacteria seems to actually benefit from recognition by, rather than evasion from, human T cell immunity (62, 63). Nevertheless, we still believe that our scoring system can be useful to identify potentially immunodominant Mtb-derived epitopes, because the Mtb-derived HLA-I and HLA-II epitopes with the highest scores (KLAGGVAVI and DWYSPACGKAGCQTYKWETF), derived from the 65 kDa heat shock protein and the antigen 85B, respectively, have previously been described as potently immunodominant epitopes (64, 65). Further characterization of T cell reactivity for Mtb epitopes with high and low immunogenicity scores may unveil critical insights into the uniqueness of anti-mycobacterial T cell immunity.

One remarkable feature of our immunogenicity score system is that the score dynamics between single-aa mutants are, at least to some extent, predictive of the mutational impacts on immunogenicity. In order to drive potent and sustainable immunity against highly mutable pathogens such as HIV and HCV, the criteria of high immunogenicity may not be sufficient because of potential escape mutations. By simulating all possible single amino acid variants, our framework allows us to estimate the potential impacts of such escape mutations. It should be noted that this all-mutant strategy leads to overestimation of escape potential, because, in a real-world, some of the amino acid variations are unfeasible due to many factors such as codon usage patterns in the genome of the target organism, decreased replication fitness, loss-of-function of essential enzymes. However, since our primary interest is to obtain epitopes with relatively high immunogenicity and yet with relatively low escape potential, overestimation of escape potential may not be a critical problem. In contrast, underestimation of escape potential could lead to failure of filtering out candidates that may lose their immunogenicity by only a single mutation. Practically, the proposed two-metrics-based approach of epitope prioritization could expedite the development of vaccines applicable to a wide range of individuals, and could also improve the predictability of the responsiveness to checkpoint blockade immunotherapies when combined with known biomarkers such as tumor mutational burden (66–68).

There are indeed several caveats to be noted in this study in addition to those discussed in the above sections. First, our current model may be suboptimal because only a small fraction of possible sequence space is experimentally covered so far, and there is considerable heterogeneity of the experimental methodologies employed. T cell-based assays of identical readouts (e.g., IFN-γ secretion) using cells obtained from multiple individuals for all candidate epitopes and their single amino acid variants would be ideal for defining a universal scale of the probability of T cell reactivity through machine learning. Second, due to the lack of sufficient annotations, we did not distinguish TCR repertoires from different T cell subsets such as CD8^+^ and CD4^+^ T cells with either pro-inflammatory or regulatory functions. Since the dynamics of TCR repertoires in these subsets are distinct, utilization of subset-specific TCR repertoire may further improve the predictability of epitope immunogenicity. Third, in a real-world setting *in vivo*, we need to take into consideration the spatiotemporal proximity of multiple epitopes simultaneously presented at the focus of inflammation. In such circumstances, the “threshold” of epitope immunogenicity itself is likely altered, and bystander activation also likely contributes to the gross immune outcome. Nevertheless, it is encouraging that the proposed framework predicted many epitopes consistently with previous experimental studies. Further validations both *in vitro* and *in vivo* (in animal models and from observational studies in humans) are warranted. Elucidation of the pan-epitope principles of epitope targeting by human T cell immunity would have significant clinical and biological implications, and we hope that our study serves as a starting point for future investigations.

## Materials and Methods

### Computational Analysis

All computational analyses were conducted using R ver. 3.5.0 (https://www.r-project.org/) (69). The latest versions of R packages were consistently used. Compiled datasets and essential in-house functions are available as the R package *Repitope* on GitHub (https://github.com/masato-ogishi/Repitope). Other scripts are available upon request.

### Peptide Sequence Datasets

HLA-I-restricted peptide sequences of 8-aa to 11-aa lengths with T cell assay results were collected from public databases [Immune Epitope Database (IEDB, as of May 7th, 2018) (42), the best-characterized CTL epitopes from Los Alamos National Laboratory (LANL) HIV Sequence Database (38), LANL HCV Sequence Database (36), EPIMHC (40), MHCBN (37), and TANTIGEN (39)], and previous publications (34, 35, 41). HLA-II-restricted peptide sequences of 11-aa to 30-aa lengths with T cell assay results were collected from the IEDB database (as of May 7th, 2018). As for peptides retrieved from the IEDB database, only those with evidence of functional T cell response were included. The list of evidence included is as follows: activation, antibody help, CCL2/MCP-1 release, CCL3/MIP-1a release, CCL4/MIP-1b release, CCL5/RANTES release, CXCL10/IP-10 release, CXCL9/MIG release, cytotoxicity, decreased disease, degranulation, disease exacerbation, GM-CSF release, granulysin release, granzyme A release, granzyme B release, IFNg release, IL-10 release, IL-12 release, IL-13 release, IL-17 release, IL-17A release, IL-1b release, IL-2 release, IL-21 release, IL-22 release, IL-23 release, IL-3 release, IL-4 release, IL-5 release, IL-6 release, IL-8 release, lymphotoxin A/TNFβ release, pathogen burden after challenge, perforin release, proliferation, protection from challenge, survival from challenge, T cell- APC binding, T cell help, TGFβ release, TNF release, TNFα release, tolerance, tumor burden after challenge, and type IV hypersensitivity (DTH). Peptides presented on non-human MHC molecules were discarded, whereas those presented on HLA molecules in non-human hosts (e.g., transgenic mice) were included. Peptides retrieved from LANL and TANTIGEN databases were *a priori* assumed to be immunogenic. In this manner, 21,162 and 31,693 HLA-I and HLA-II-restricted peptides were identified, respectively, of which 1,873 (8.9%) and 4,505 (14.2%) had contradicting annotations on immunogenicity. In such cases, peptides with at least one positive annotation were considered immunogenic, given that coexistence of negative assay results does not necessarily preclude the possibility of being recognized by any of the TCRs at a population-level. Eventually, 6,957 (32.9%) and 16,642 (52.5%) HLA-I and HLA-II-restricted peptides were considered immunogenic, respectively. Collected peptide sequences and annotations are summarized in Supplementary Data Sheet 2.

Sequences of peptides restricted on non-human MHC molecules were collected from the IEDB database (as of as of May 7th, 2018) as described above. The following species were considered primate: bonobo, chimpanzee, gorilla, marmoset, and rhesus macaque. Meanwhile, mouse and rat were considered rodent. Eventually, 411 and 8,756 MHC-I-restricted peptides, and 76 and 8,445 MHC-II-restricted peptides were identified for primates and rodents, respectively. Collected peptide sequences and annotations can be found in Supplementary Data Sheet 2.

### TCR Sequence Datasets

TCR repertoire datasets were collected from the NCBI Sequence Read Archive (SRA) and a previous study led by Britanova et al. (21). Repertoire datasets derived from healthy donors were searched through SRA, and the following BioProjects were included: PRJNA389805, PRJNA329041, PRJNA273698, PRJNA258001, PRJNA229070, PRJNA79707, and PRJNA79435. Also, pooled sequence data from 39 healthy donors were retrieved from the paper by Britanova et al. In this manner, a total of 206 datasets, of which only five were pooled datasets, were collected. Pooled and individual datasets were derived from a total of 561 and 103 healthy donors, respectively. Fastq files were obtained using fastq-dump script with the following options: –gzip –skip-technical –readids –read-filter pass –dumpbase –split-files –clip –accession [SRA run number]. A total of 23,006,555 CDR3β sequences were extracted using MiXCR software (43).

Public TCR clonotypes, the ones commonly observed in multiple datasets, were identified from the pool of CDR3β sequences mentioned above. Britanova et al. (21) reported that 10,691 clonotypes were shared between at least six of 39 (15.4%) donor-derived top 100,000 clonotype sets. Based on their findings, we decided to extract CDR3β sequences identified in at least 22 out of 206 (10.8%) different datasets, and we subsequently identified 191,326 (0.83%) public clonotypes. A randomly sampled 10,000 clonotypes were used for TCR-peptide contact potential profiling.

### TCR-Peptide Contact Potential Profiling

T cell epitopes presented on the MHC molecules must be recognized by the TCRs of CD8^+^ CTLs and CD4^+^ Th cells with sufficiently high affinity to trigger subsequent immunological cascades. Undoubtedly, the vast majority of TCR repertoire is not involved in recognition of any given peptide. Moreover, considering the substantial conformational flexibility observed upon binding of TCRs to pMHC complexes, one can assume that only a subset of residues of the peptide and the epitope-recognition domain, namely, the CDR3β loop, serve as a “seed” of intermolecular docking during the early phase of molecular scanning (29, 32). With these in mind, identifying the best contact site between a given pair of peptide and CDR3β sequences is conceptually similar to solving a local pairwise sequence alignment problem. One caution is that higher scores must be given to more strongly interacting residue pairs instead of more biochemically similar residue pairs as opposed to ordinary alignments. For this purpose, amino acid pairwise contact potential (AACP) scales from the AAIndex database (70) (http://www.genome.jp/aaindex/AAindex/list_of_potentials) were adopted to generate custom substitution matrices. As stronger interresidue interactions yield smaller free energy values (negative numbers), and as the Smith-Waterman local alignment algorithm attempts to maximize the alignment score of a given sequence (TCR fragment) against the whole target sequence (peptide), the optimal pairwise alignment using a custom substitution matrix derived from the sign-inverted version of a pairwise contact potential scale would in principle correspond to the best intermolecular contact. For comparative purposes, both inverted and non-inverted versions were tested. Values were rescaled to a range from zero to one for subsequent analyses. A set of non-inverted, non-rescaled AACP scales is provided as Supplementary Data Sheet 6. For repertoire-wide CPP analysis, the 191,326 pooled public CDR3β sequences were randomly down-sampled to 10,000 sequences with the relative abundance ratios retained. The sequences were then fragmented by a sliding window strategy to generate a fragment library. Since the interacting orientations (either forward parallel or antiparallel) are unknown for most of the cases, reversed CDR3β sequences were also fragmented and combined. The sizes of fragments were from 3-aa to 8-aa and from 3-aa to 11-aa for MHC-I and MHC-II predictions, respectively. For single-TCR CPP (sCPP) analysis, every single TCR instead of public TCR repertoire was used to generate a fragment library. To perform sequence alignments, the *pairwiseAlignment* function implemented in the *Biostrings* package in *Bioconductor* (https://www.bioconductor.org/) (71) was utilized. This function seeks an optimal alignment which maximizes the overall alignment score defined as a sum of pairwise scores. Alignment type was set “global-local” to obtain an optimal alignment of a given set of TCR fragments against the consecutive subsequences of any given peptide. Gaps were not allowed. A set of alignment scores was summarized by calculating representative statistics. Following statistics were calculated using the functions implemented in the *psych* package: mean (Mean), standard deviation (SD), standard error of the mean (SE), median (Med), median absolute deviation (MAD), interquartile range (IQR), 10% quantile (Q10), 90% quantile (Q90), skewness (Skew), and kurtosis (Kurt). Predictive features were generated by combining the fragment length, the AACP scale, and the type of statistics. In this manner, 700 CPP features per one fragment length were generated for each of the peptides.

### Peptide Descriptors

Apart from CPP features, sequence-based estimates of physicochemical properties were also calculated. Each peptide sequence was converted into a set of consecutive fragments of a defined amino acid length, and peptide descriptors were calculated against each of the fragments using functions in the *Peptide* package. Following functions were utilized: *aIndex, blosumIndices, boman, charge, crucianProperties, fasgaiVectors, hmoment, hydrophobicity, instaIndex, kideraFactors, mswhimScores, pI, protFP, vhseScales*, and *zScales*. The distributions of the values were summarized similarly to CPP features. Additionally, 20 binary features indicating whether the peptide of interest is free from a specific amino acid residue were included. The peptide length was also included as a feature.

### Feature Selection

Preprocessing followed by importance-based feature selection was repeated ten times with different random seeds. The analysis workflow consisted of the following steps. First, the peptide set was randomly split in a ratio of 4:1 for the training and testing subdatasets. Second, using the training subdataset, a preprocessing function was defined; numerical features were centered and scaled; a peptide length and binary features representing existence or absence of each residue were left unaltered. The same preprocessing filter designed from the training subdataset was also applied to the testing subdataset. Third, highly correlated features were removed, with the threshold of correlation coefficient being 0.75. Fourth, features were filtered based on the importance values calculated using the *generateFilterValuesData* function with the random forest (RF) method (*randomForestSRC.rfsrc*) implemented in the *mlr* package (72). The 100 most important features were retained unless otherwise stated. In the first five repeats, the names of the peptide descriptors and the CPP features were ordered in ascending order. In the latter five repeats, on the other hand, the names of those features were ordered in descending order. In this manner, the inherent bias of feature selection owing to the lexicographical order of the names of features was avoided. Finally, features kept in all the ten repeats were defined as the most predictive features and were utilized hereafter.

### TCR-pMHC Structure Analysis

The structures of TCR-pMHC complexes with known experimentally determined affinities were collected manually from the literature using the ATLAS database (https://zlab.umassmed.edu/atlas/web/) (73). A total of 95 structures were retrieved, of which 82 contained non-mutated (wildtype) TCRs. Contact sites were identified using the PRODIGY server (26, 27). TCR contact footprints were defined as the distributions of the numbers of contacts at each of the peptide positions.

For the analysis of correlation with affinities, sCPP features were computed as described in the above sections, and the single best feature was selected from a univariate analysis for each of the AAIndex scales. Multivariate regression with stepwise feature selection was conducted using the *stepAIC* function implemented in the *MASS* package. The variance inflation factors (VIFs) were computed using the *vif* function implemented in the *car* package.

Sequence permutation experiments were performed as follows. Peptide sequences were replaced with a set of random sequences of the same lengths. In contrast, because of the fragmentation strategy employed, a simple replacement would lead to incomplete disruption of hidden but essential motifs. Therefore, we instead replaced the TCR-derived fragment library with a randomly chosen set of random fragments that do not overlap with the original fragment library. Permutation experiments were iterated 1,000 times.

### HLA Binding Prediction

Predicted binding strength against twelve representative HLA-I alleles (A^*^01:01, A^*^02:01, A^*^03:01, A^*^24:02, A^*^26:01, B^*^07:02, B^*^08:01, B^*^27:05, B^*^39:01, B^*^40:01, B^*^58:01, and B^*^15:01) from NetMHC 4.0 (74) and six representative HLA-II alleles (DRB1^*^0101, DRB3^*^0101, DRB4^*^0101, DRB5^*^0101, DPA1^*^0103-DPB1^*^0101, and DQA1^*^0101-DQB1^*^0201) from NetMHCIIpan 3.2 (56) for MHC-I and MHC-II immunogenicity prediction, respectively, were incorporated in the machine learning process for immunogenicity prediction because the stability of the peptide-MHC complex is a known correlate of immunogenicity (75). Default parameters were used for prediction. Thresholds of percentile ranks for strong and weak binders were set at 0.5 and 2% in MHC-I, and 2 and 10% in MHC-II, respectively.

### Machine Learning

The most predictive peptide descriptors and CPP features, with or without predicted HLA bindings, were compressed into a linear coordinate system through machine learning. We utilized extremely randomized trees (ERT) algorithm implemented in the *extraTrees* package (76) because of its computational efficiency and robustness against overfitting. The model-specific parameter *mtry* was set 5. Other hyperparameters were set as defaults. Class weights were provided to compensate for imbalanced class distributions. Probabilistic estimates were computed by conducting five times repeated 5-fold cross-validations (CVs). This strategy ensures that any peptide is subjected to five times repeated predictions by models trained from a set of peptides that do not contain the peptide in question. The averaged probability estimates were termed “immunogenicity scores.” Unless otherwise stated, we utilized immunogenicity scores from machine learning without predicted HLA binding-based features. For peptides not included in our epitope dataset, immunogenicity scores are defined as the averaged probability estimates of the 25 ERT models generated during CVs. Prediction variances were evaluated by computing coefficients of variance which are defined as standard deviations divided by means.

The IEDB Epitope Cluster Analysis Tool (http://tools.iedb.org/cluster/) was utilized to test the effect of sequence-level peptide homology on immunogenicity prediction. Clustering was performed with the homology threshold of 80%. When clustering filter was applied, only a single peptide was randomly chosen from each of the clusters before feature preprocessing in the machine learning workflow.

### Neighbor Network

Single amino acid mutations, namely, substitutions, insertions, and deletions, have multifaceted effects on the immunogenicity of MHC-presented peptides. To systematically analyze their effects on immunogenicity, we adopted a network-style representation of the epitope data structures, termed a “neighbor network,” where pairs of peptide sequences (nodes) with just one edit distance were defined as “neighbors” and regarded as edges. Each edge was directed from the peptide with a lower immunogenicity score to the peptide with a higher score. Edge weight was defined as the ratio of the lower score to the higher score so that less immunogenically similar peptides were mapped more distant from each other in sequence space due to the smaller edge weight. Clustering was performed using a walktrap algorithm implemented in the *igraph* package (77). For each cluster, sequences were aligned using the ClustalW algorithm implemented in the *msa* package (78). A consensus sequence was generated using the method implemented in the *Biostrings* package with an ambiguity threshold of 0.5. Gaps were treated equally to ambiguities. Gaps/ambiguities were expressed as “X.”

### *In silico* Mutagenesis

For a given “parental” peptide, the neighbor network was computationally expanded by simulating all possible single amino acid substituted mutant peptides. Insertions and deletions were not taken into consideration. These artificially introduced mutations could affect various aspects of T cell immunity, including aberrant proteolytic cleavage and altered MHC presentation, these possibilities were not rigorously examined since our primary interest is on exploring the net immunogenicity of mutated peptides if appropriately presented and scanned by TCRs. Immunogenicity scores for simulated peptides were computed by extrapolation using the classifiers trained using all experimentally annotated data. Neighbor network analysis was conducted as described. Extrapolated immunogenicity scores of all neighbors and neighbors residing in the same cluster with the parental peptide were averaged to yield overall-mean and cluster-mean immunogenicity scores, respectively.

### Immune Transition

Loss of immunogenicity due to mutation is also called escaping, evasion, and sometimes immunoediting. In the present study, we refer to this phenomenon as escaping. In contrast, there is no appropriate terminology for the mutation-driven acquisition of immunogenicity, and thus we refer to this phenomenon as “epitope formation.” For the sake of simplicity, we do not take into consideration epitope-extrinsic mechanisms such as impaired intracellular antigen processing, somatic loss of HLA heterozygosity (79, 80), and checkpoint-mediated T cell exhaustion and apoptosis (81) in this work. Escaping and epitope formation due to mutations can be understood as two sides of the same coin in sequence space, and therefore, we propose a higher-order concept, “immune transition,” which is defined as a change in immunogenicity between any MHC-presented peptide and its single amino acid variant. Note that the synergistic effects of two or more mutations on the changes in immunogenicity are beyond the scope of the present study.

To further characterize the theoretical boundary of immunogenic and non-immunogenic peptides, we categorized peptides with evidence of immune transition found in our dataset as “transitional” and analyzed separately. Note that we cannot certainly define “non-transitional” peptides, as the lack of immune transition in our dataset may merely reflect the lack of experimental evaluation. For transitional peptides, *in silico* mutagenesis followed by neighbor network analysis was conducted. Mean immunogenicity scores of both all single amino acid mutants and those within the same cluster to the parental peptide were computed.

### Immunogenicity Score Dynamics

For quantitative comparison of immunogenicity for a given pair of peptides, either the ratio of immunogenicity scores (divided by the lower score), termed “relative score change,” or the absolute difference of immunogenicity scores divided by the lower score, termed “normalized score change,” was used as a metric. To systematically explore the position-specific mutational impacts for a given parental peptide, relative score change was computed for every pair of computationally simulated mutants. For heatmap analysis, peptide pairs were grouped based on their mutated residue combinations, and median normalized score changes were calculated for each group. Heatmaps were visualized using the *ComplexHeatmap* package (82).

### Escape Potential

Escape potential of a given peptide was defined as the maximum difference of the cluster-mean immunogenicity scores between the cluster to which the target peptide belongs and the other clusters in the neighbor network constructed from all possible single amino acid variants assigned with simulated immunogenicity scores. A large escape potential means that a single mutation could cause a significant loss of predicted immunogenicity. A negative value indicates that the target peptide resides in the least immunogenic cluster, and the target epitope would not likely lose its immunogenicity further by any single mutation.

## Author Contributions

MO and HY conceived and discussed the concept. MO designed the entire study, performed computational analyses, and drafted the manuscript. MO and HY wrote the manuscript.

### Conflict of Interest Statement

The authors declare that the research was conducted in the absence of any commercial or financial relationships that could be construed as a potential conflict of interest.
