# Supplementary material for: Quantitative Prediction of the Landscape of T Cell Epitope Immunogenicity in Sequence Space
Source: Front Immunol. 2019 Apr 16;10:827. doi: 10.3389/fimmu.2019.00827 (PMC6477061; doi:10.3389/fimmu.2019.00827)
Supplement: Supplementary file 1 [file Presentation_1.pdf]

Supplementary Material

Supplementary Figures

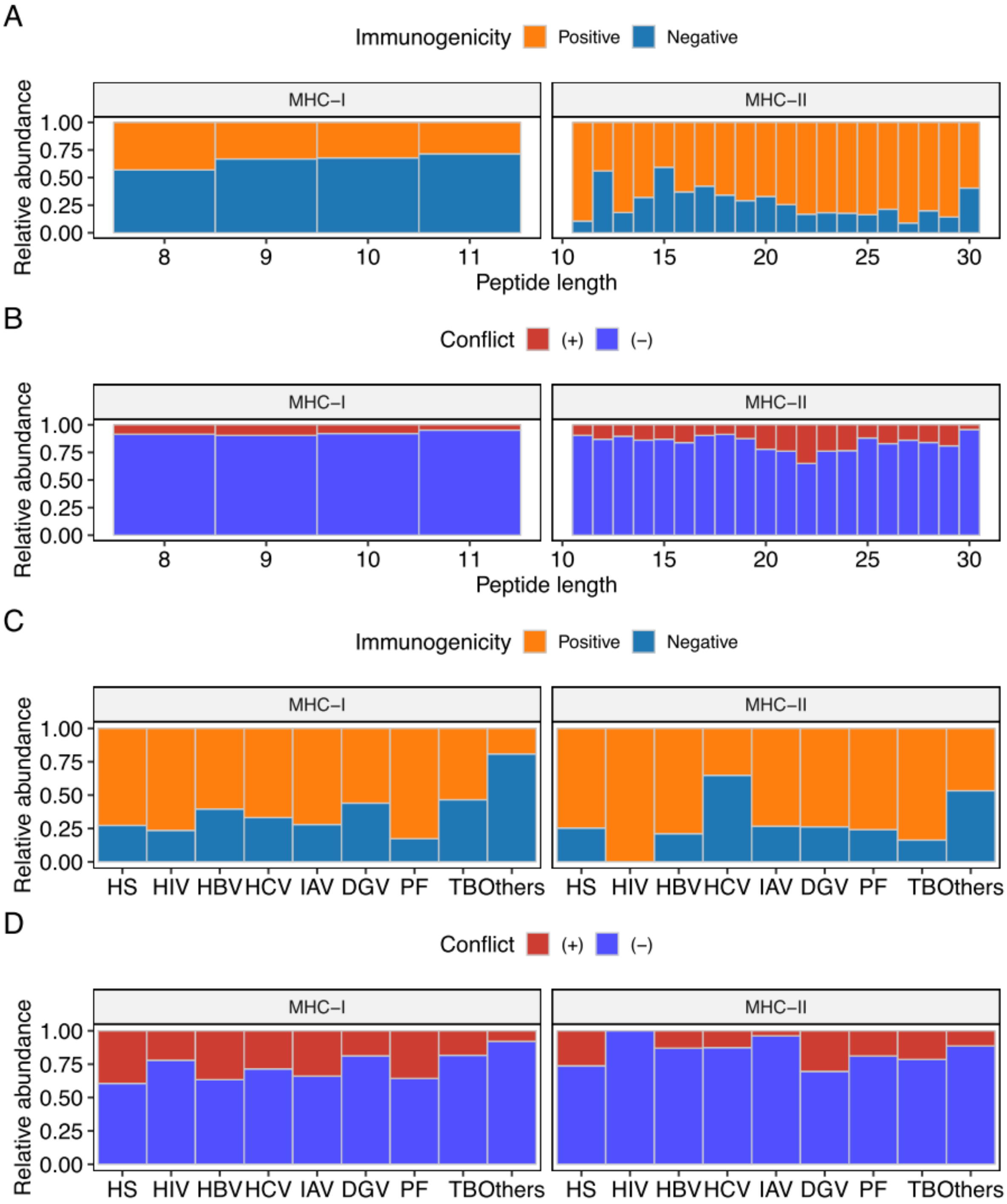

Supplementary Figure 1. Characteristics of the epitope dataset. (A and B) Relative abundance of (A) immunogenicity annotations and (B) conflicts in immunogenicity annotations among peptides of different lengths. (C and D) Relative abundance of (C) immunogenicity annotations and (D) conflicts in immunogenicity annotations among peptides of different origins. HS, homo sapiens; HIV, human

immunodeficiency virus type 1; HBV, hepatitis B virus; HCV, hepatitis C virus; IAV, influenza A virus; DGV, dengue virus; PF, *Plasmodium falciparum*; TB, *Mycobacterium tuberculosis*; Others, peptides derived from other organisms (peptides with no source organism annotation were excluded).

A

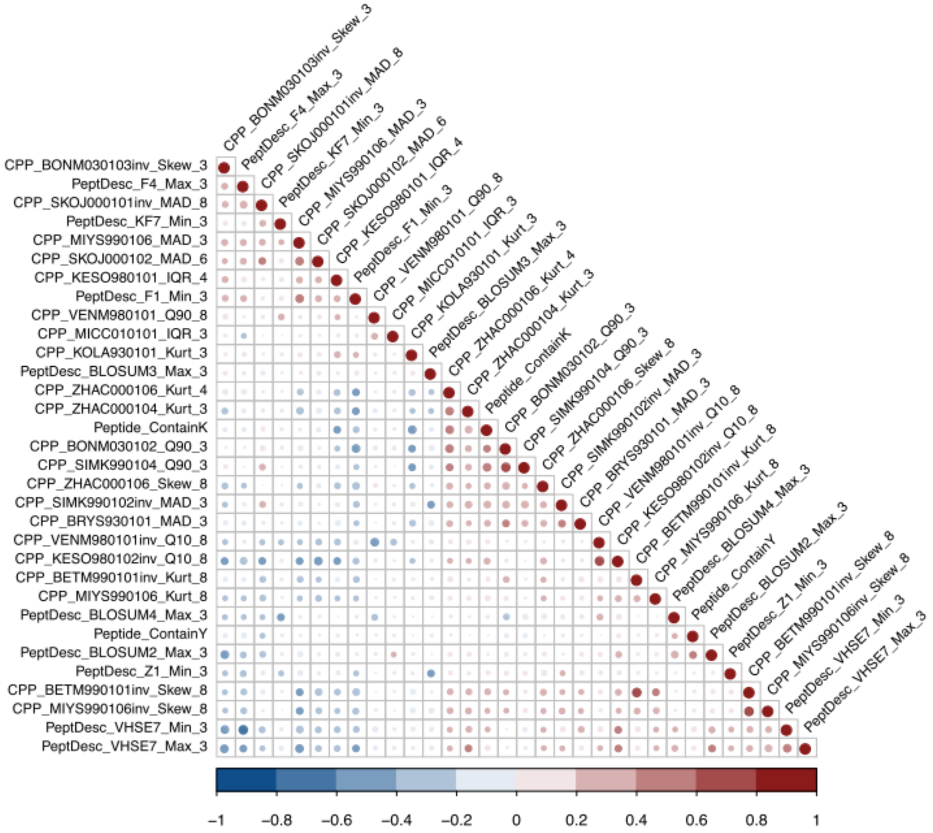
CPP_BONM030103inv_Skew_3
PeptDesc_F4_Max_3
CPP_SKOJ000101inv_MAD_8
PeptDesc_KF7_Min_3
CPP_MIYS990106_MAD_3
CPP_SKOJ000102_MAD_6
CPP_KESO980101_IQR_4
PeptDesc_F1_Min_3
CPP_VENM980101_Q90_8
CPP_MICC010101_IQR_3
CPP_KOLA930101_Kurt_3
PeptDesc_BLOSUM3_Max_3
CPP_ZHAC000106_Kurt_4
CPP_ZHAC000104_Kurt_3
Peptide_ContainK
CPP_BONM030102_Q90_3
CPP_SIMK990104_Q90_3
CPP_ZHAC000106_Skew_8
CPP_SIMK990102inv_MAD_3
CPP_BRYS930101_MAD_3
CPP_VENM980101inv_Q10_8
CPP_KESO980102inv_Q10_8
CPP_BETM990101inv_Kurt_8
CPP_MIYS990106_Kurt_8
PeptDesc_BLOSUM4_Max_3
Peptide_ContainY
PeptDesc_BLOSUM2_Max_3
PeptDesc_Z1_Min_3
CPP_BETM990101inv_Skew_8
CPP_MIYS990106inv_Skew_8
PeptDesc_VHSE7_Min_3
PeptDesc_VHSE7_Max_3
−1
−0.8
−0.6
−0.4
−0.2
0
0.2
0.4
0.6
0.8
1

B

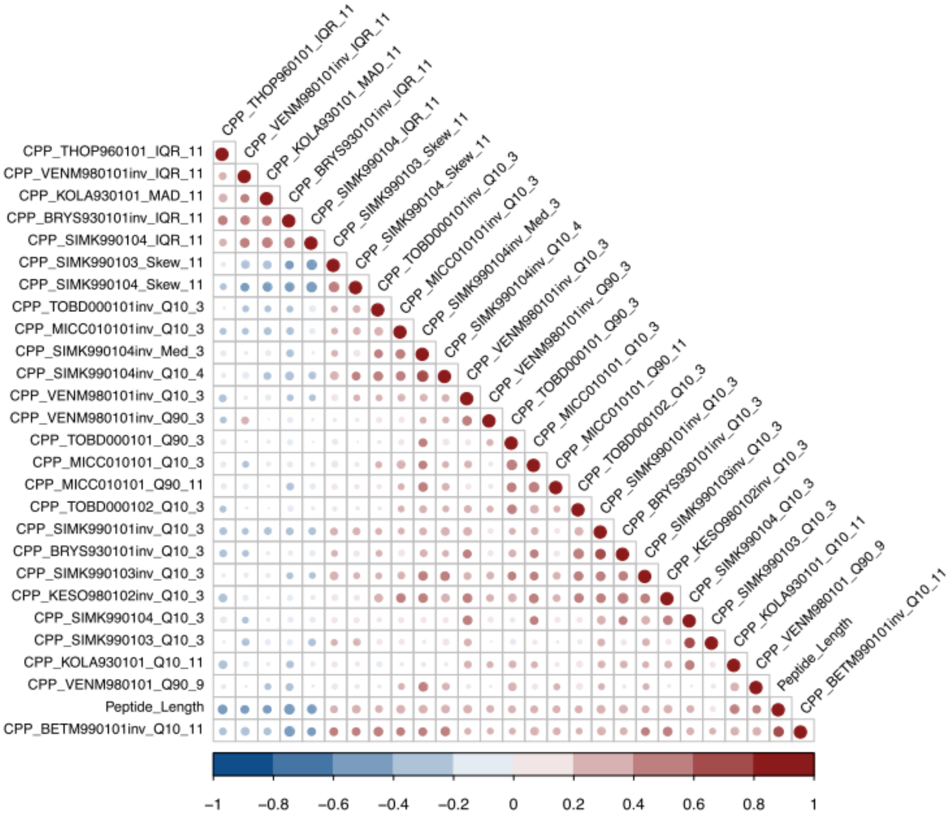
CPP_THOP960101_IQR_11
CPP_VENM980101inv_IQR_11
CPP_KOLA930101_MAD_11
CPP_BRYS930101inv_IQR_11
CPP_SIMK990104_IQR_11
CPP_SIMK990103_Skew_11
CPP_SIMK990104_Skew_11
CPP_TOBD000101inv_Q10_3
CPP_MICC010101inv_Q10_3
CPP_SIMK990104inv_Med_3
CPP_SIMK990104inv_Q10_4
CPP_VENM980101inv_Q10_3
CPP_VENM980101inv_Q90_3
CPP_TOBD000101_Q90_3
CPP_MICC010101_Q10_3
CPP_MICC010101_Q90_11
CPP_TOBD000102_Q10_3
CPP_SIMK990101inv_Q10_3
CPP_BRYS930101inv_Q10_3
CPP_SIMK990103inv_Q10_3
CPP_KESO980102inv_Q10_3
CPP_SIMK990104_Q10_3
CPP_SIMK990103_Q10_3
CPP_KOLA930101_Q10_11
CPP_VENM980101_Q90_9
Peptide_Length
CPP_BETM990101inv_Q10_11
−1
−0.8
−0.6
−0.4
−0.2
0
0.2
0.4
0.6
0.8
1

Supplementary Figure 2. Correlograms of the minimal sets of features selected as the most predictive of epitope immunogenicity. Eighty percent of the entire epitope dataset was randomly extracted, and feature importance was estimated by training a random forest classifier. Based on the feature importance estimates, top 100 features were selected. This selection process was repeated ten times, and the consensus feature sets were generated for both MHC-I and MHC-II epitopes. Correlations between each of the features are depicted as correlograms. Features computed through the TCR-peptide contact potential profiling (CPP) have the suffix of "CPP_." computed from peptide descriptors analysis have the suffix of "PeptDesc_." (A) Features for MHC-I-restricted peptides (N=32). (B) Features for MHC-II-restricted peptides (N=27). Features were reordered by the hierarchical clustering algorithm.

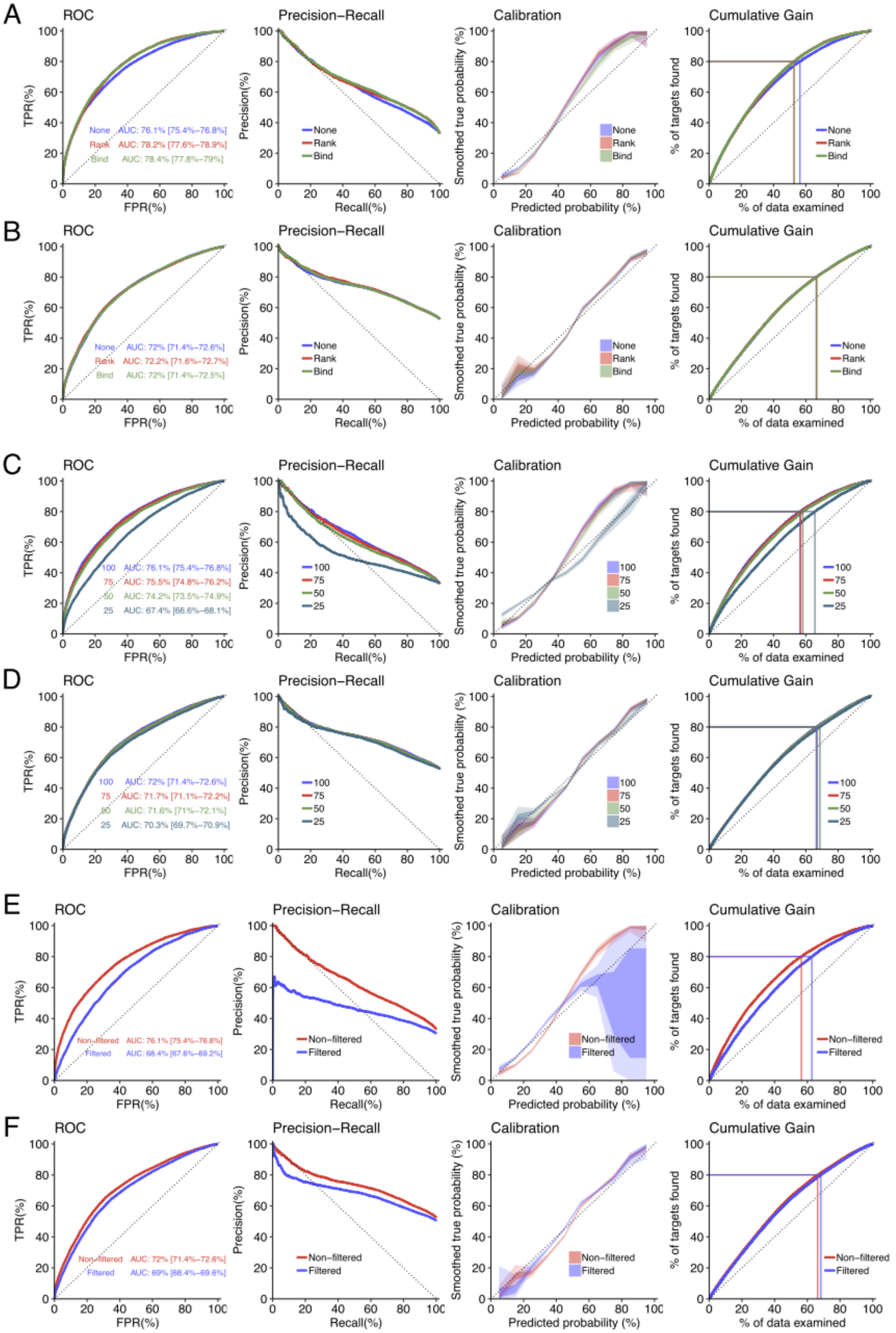
A
ROC
Precision–Recall
Calibration
Cumulative Gain
TPR(%)
FPR(%)
None AUC: 76.1% [75.4%–76.8%]
Rank AUC: 78.2% [77.6%–78.9%]
Bind AUC: 78.4% [77.8%–79%]
Precision(%)
Recall(%)
Smoothed true probability (%)
Predicted probability (%)
% of targets found
% of data examined
None
Rank
Bind
B
None AUC: 72% [71.4%–72.6%]
Rank AUC: 72.2% [71.6%–72.7%]
Bind AUC: 72% [71.4%–72.5%]
C
100 AUC: 76.1% [75.4%–76.8%]
75 AUC: 75.5% [74.8%–76.2%]
50 AUC: 74.2% [73.5%–74.9%]
25 AUC: 67.4% [66.6%–68.1%]
100
75
50
25
D
100 AUC: 72% [71.4%–72.6%]
75 AUC: 71.7% [71.1%–72.2%]
50 AUC: 71.6% [71%–72.1%]
25 AUC: 70.3% [69.7%–70.9%]
E
Non–filtered AUC: 76.1% [75.4%–76.8%]
Filtered AUC: 68.4% [67.6%–69.2%]
Non–filtered
Filtered
F
Non–filtered AUC: 72% [71.4%–72.6%]
Filtered AUC: 69% [68.4%–69.6%]

Supplementary Figure 3. Robustness analysis of immunogenicity predictions. Performances of probabilistic estimates computed through machine learning under various conditions were examined. (A and B) Addition of MHC binding prediction results as features for machine learning. Rank, percentile ranks; Bind, binding strength categories. (A) MHC-I-restricted peptides. (B) MHC-II-restricted peptides. (C and D) Machine learning with smaller cutoff values for feature selection. (C) MHC-I-restricted peptides. The actual numbers of features retained after feature selection were 32, 26, 19, and 7, respectively. (D) MHC-II-restricted peptides. The actual numbers of features retained were 27, 23, 21, and 12, respectively. (E and F) Peptide homology-based clustering before machine learning. The IEDB Epitope Cluster Analysis Tool (**http://tools.iedb.org/cluster/**) was used to cluster the peptides with the homology threshold of 80%. One peptide per cluster was randomly chosen ("Filtered"). This step is intended to reduce the effect of potential overfitting due to sequence-level homology. (E) MHC-I-restricted peptides. A total of 16,765 peptides were retained after clustering. (F) MHC-II-restricted peptides. A total of 23,983 peptides were retained after clustering.

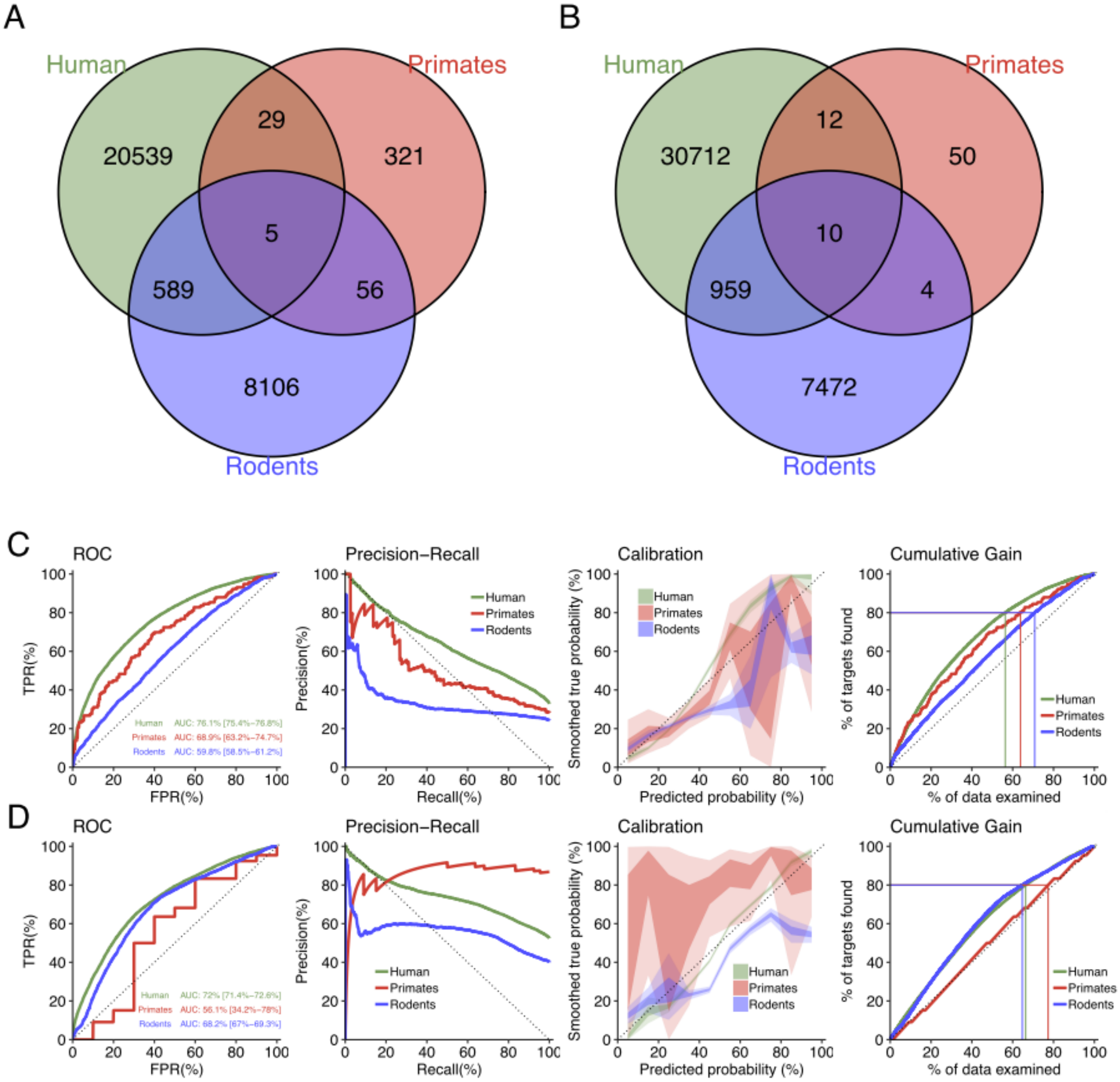

Supplementary Figure 4. Extrapolation of the human immunogenicity prediction framework for primate and rodent epitopes. (A and B) Overlaps between (A) MHC-I-restricted and (B) MHC-II-restricted peptides presented on MHC molecules of different species. (C and D) Predictive performances of the probabilities of immunogenicity for (C) MHC-I-restricted and (D) MHC-II-restricted peptides. Probabilities for non-human peptides were estimated by applying the classifiers trained on the human peptide datasets.

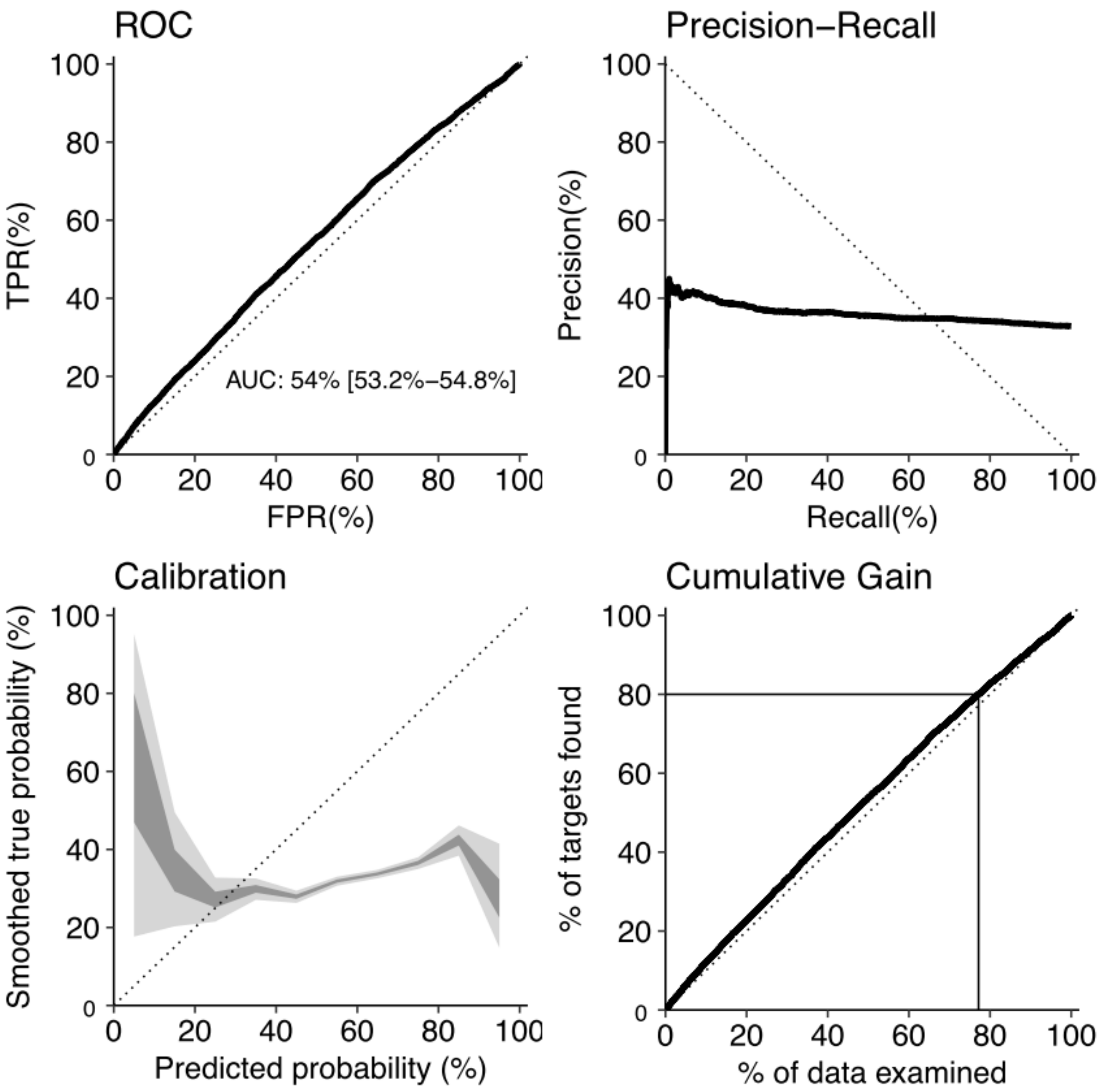

Supplementary Figure 5. Immunogenicity prediction of HLA-I-restricted peptides by the IEDB Class I Immunogenicity Tool. Classification performances were evaluated using the prediction scores (rescaled to 0-1 range) derived from the IEDB Class I Immunogenicity Tool (34) (**http://tools.iedb.org/immunogenicity/**) with default settings. Note that peptides used for training the model in the original paper were not excluded from prediction, which theoretically overestimates its predictive performance.

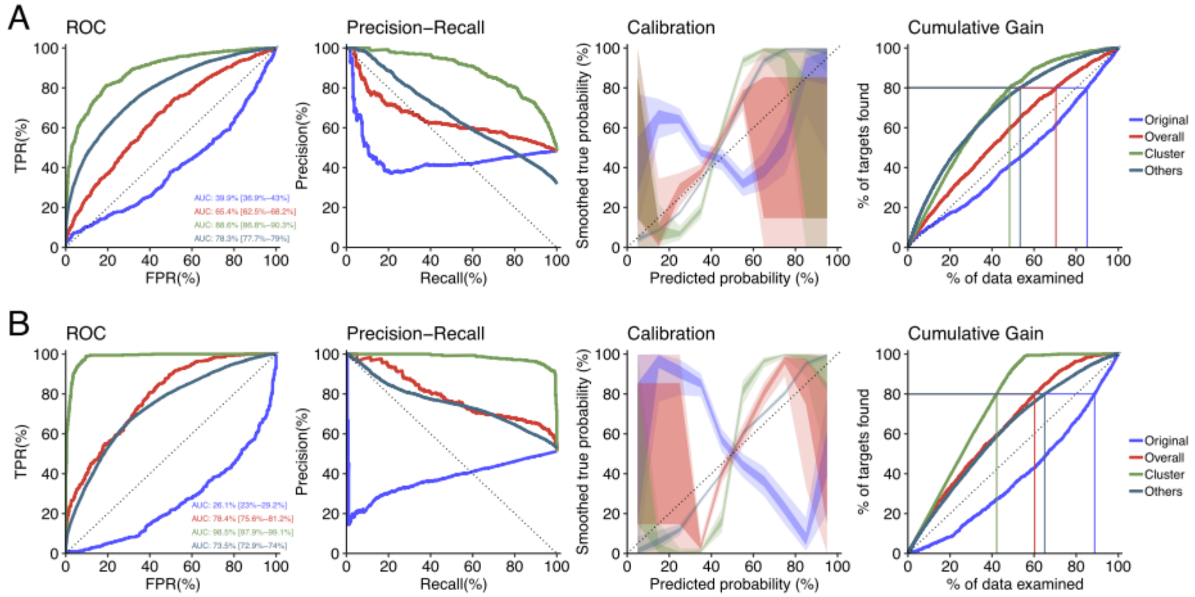

Supplementary Figure 6. Improvement of predictive performances of immunogenicity scores for transitional peptides by integrating the neighbor network architectures in sequence space. Predictive performances of the immunogenicity scores of (A) MHC-I- and (B) MHC-II-restricted transitional peptides, *i.e.*, peptides with at least one neighbor of opposite immunogenicity annotation in our dataset, were evaluated. We identified 1,360 and 976 transitional peptides for MHC-I and MHC-II, respectively. We expanded their neighbor networks by computing immunogenicity scores for 232,723 and 292,437 all possible single-aa mutants of transitional MHC-I- and MHC-II-restricted peptides, respectively. Original, original immunogenicity scores; Overall, mean immunogenicity scores of all neighbors. Cluster, mean immunogenicity scores of neighbors assigned to the cluster containing the parent peptide. Others, original immunogenicity scores for peptides with no evidence of immune transition (shown for comparison purpose).

Supplementary Tables

Supplementary Table 1. Variance inflation factors (VIFs) of the features used in the multivariate regression analysis against experimentally determined TCR affinities.

Feature	VIF
sCPP_BETM990101inv_Skew_4	1.25
sCPP_KESO980102inv_Kurt_3	1.11
sCPP_KOLA930101_Skew_4	1.29
sCPP_SIMK990104_SD_3	1.14
sCPP_VENM980101inv_Min_3	1.06

Supplementary Datasets

Supplementary Data 1. (separate file)

Affinities and structures of known TCR-peptide pairs collected from the literature

Supplementary Data 2. (separate file)

Human, primate, and rodent T cell epitope data sets.

Supplementary Data 3. (separate file)

Feature importance estimates for MHC-I and MHC-II epitope immunogenicity prediction.

Supplementary Data 4. (separate file)

Immunogenicity scores for MHC-I and MHC-II epitope.

Supplementary Data 5. (separate file)

Single amino acid mutant pairs identified in the human MHC-I and MHC-II epitope datasets.

Supplementary Data 6. (separate file)

A list of AAIndex amino acid pairwise contact potential scales utilized in this study.
